# A Novel Small Molecule Inhibitor of Influenza A Viruses that Targets Polymerase Function and Indirectly Induces Interferon

**DOI:** 10.1371/journal.ppat.1002668

**Published:** 2012-04-26

**Authors:** Mila Brum Ortigoza, Oliver Dibben, Jad Maamary, Luis Martinez-Gil, Victor H. Leyva-Grado, Pablo Abreu, Juan Ayllon, Peter Palese, Megan L. Shaw

**Affiliations:** 1 Department of Microbiology, Mount Sinai School of Medicine, New York, New York, United States of America; 2 Department of Medicine, Mount Sinai School of Medicine, New York, New York, United States of America; National Institutes of Health, United States of America

## Abstract

Influenza viruses continue to pose a major public health threat worldwide and options for antiviral therapy are limited by the emergence of drug-resistant virus strains. The antiviral cytokine, interferon (IFN) is an essential mediator of the innate immune response and influenza viruses, like many viruses, have evolved strategies to evade this response, resulting in increased replication and enhanced pathogenicity. A cell-based assay that monitors IFN production was developed and applied in a high-throughput compound screen to identify molecules that restore the IFN response to influenza virus infected cells. We report the identification of compound ASN2, which induces IFN only in the presence of influenza virus infection. ASN2 preferentially inhibits the growth of influenza A viruses, including the 1918 H1N1, 1968 H3N2 and 2009 H1N1 pandemic strains and avian H5N1 virus. *In vivo*, ASN2 partially protects mice challenged with a lethal dose of influenza A virus. Surprisingly, we found that the antiviral activity of ASN2 is not dependent on IFN production and signaling. Rather, its IFN-inducing property appears to be an indirect effect resulting from ASN2-mediated inhibition of viral polymerase function, and subsequent loss of the expression of the viral IFN antagonist, NS1. Moreover, we identified a single amino acid mutation at position 499 of the influenza virus PB1 protein that confers resistance to ASN2, suggesting that PB1 is the direct target. This two-pronged antiviral mechanism, consisting of direct inhibition of virus replication and simultaneous activation of the host innate immune response, is a unique property not previously described for any single antiviral molecule.

## Author Summary

Influenza viruses are rapidly developing resistance against available anti-influenza drugs and consequently there is an urgent demand for new treatment approaches. We identified compound ASN2 in a high-throughput screen for molecules that are capable of inducing the antiviral cytokine interferon (IFN) in the presence of influenza virus infection. Normally, influenza virus blocks IFN production, an activity that is dependent on the viral NS1 protein and contributes to the ability of the virus to cause disease in an infected host. We show that ASN2 is a potent inhibitor of influenza A virus and can partially protect infected animals from disease and death. ASN2 acts by targeting influenza virus polymerase function which results in inhibition of virus replication, and as a consequence, NS1 expression. Thus the ability of ASN2 to induce IFN is a “side-effect”, albeit a desirable one, of polymerase inhibition. This combination of directly inhibiting the virus while also stimulating the host immune response is a novel property for an antiviral compound.

## Introduction

Influenza viruses are members of the *Orthomyxoviridae* family [1] and are the etiological agents of influenza, a contagious, acute, and febrile respiratory disease. In the United States, seasonal influenza affects approximately 5–20 percent of the population, and influenza-related deaths range from 3,300–48,600 (average 23,600) yearly, despite the existence of vaccines and antiviral drugs [2]. The need for effective antivirals was especially apparent during the 2009 pandemic when they were used both therapeutically and prophylactically during the period before the vaccine became available [3]. This also precipitated the FDA to grant temporary emergency approval to peramivir, a neuraminidase inhibitor that is administered intravenously and therefore beneficial for treating mechanically ventilated patients [4]. Even in regular influenza seasons certain populations (such as the elderly or immunocompromised) in whom vaccination response is poor, are reliant on the availability of effective antiviral drugs to treat infections and prevent transmission.

Currently, there are two classes of FDA-approved drugs for treatment or chemoprophylaxis of influenza [5]. The M2 inhibitors, amantadine and rimantadine, block the activity of the ion channel formed by M2, and thereby prevent release of viral genome segments into the cytoplasm [6]. The rate of emergence of viruses resistant to these drugs has been increasing globally, greatly compromising their effectiveness. In fact, all currently circulating influenza A virus strains (the 2009 pandemic A/H1N1 and the seasonal A/H3N2) are resistant to M2 inhibitors [7], [8], [9], and therefore these drugs are no longer recommended for the treatment of influenza.

The other class of antiviral drugs approved for treatment of influenza A and B infections are the neuraminidase (NA) inhibitors, oseltamivir and zanamivir. NA inhibitors bind the NA protein and block its enzymatic activity, thereby preventing the efficient release of newly synthesized viruses from infected cells [1]. A rapid rise in oseltamivir resistance was seen amongst seasonal A/H1N1 isolates prior to the 2009 pandemic [10]. However, the novel pandemic A/H1N1 viruses, which have since replaced the seasonal H1N1 viruses, retain oseltamivir sensitivity. Thus, although all currently circulating influenza viruses are susceptible to inhibition with the neuraminidase inhibitors, they remain the only class of antiviral drug available for treatment of influenza infections. Therefore new antiviral strategies, including different viral targets, cellular targets or immune-modulating drugs, are sorely needed. Of those antivirals in development that act via a new mechanism, T-705 (favipiravir) has shown the most promise *in vitro* and *in vivo*, inhibiting influenza virus and other RNA viruses [11], [12]. T-705 becomes metabolized intracellularly to form T-705 ribofuranosyl triphosphate, which acts as a purine analogue and inhibits viral RNA synthesis [13].

The clinical outcome of influenza is influenced by interactions between the virus and the host, and type I interferons (IFN-α/β) are the primary mediators of the host innate immune response. These cytokines are induced following virus infection and establish an antiviral state in both infected and neighboring cells by stimulating the expression of antiviral genes known as interferon stimulated genes (ISGs). Due to the potent antiviral properties of IFN-α/β and ISGs, many viruses have evolved strategies to evade this response. Specifically, viruses often encode proteins, generically called IFN antagonists, that target pathways involved in the production and/or response to IFN-α/β [14]. For influenza A viruses the primary IFN antagonist is the NS1 protein [15], which is a multifunctional protein present at high levels in infected cells. The major role ascribed to NS1 is its ability to inhibit the IFN-α/β response at the levels of both IFNβ production and the activity of some ISGs [15], [16]. Evidence that NS1 is important for pathogenesis comes from the finding that viruses lacking a functional NS1 protein are attenuated and do not cause disease [15], [17], [18], [19], [20]. These viruses are being developed as candidates for live attenuated influenza virus vaccines [21] and by similar reasoning, NS1 therefore represents an attractive new target for influenza antiviral drugs [22].

Using a cell-based assay that monitors IFN-β induction, we screened libraries of small molecular weight compounds for their ability to restore IFN-β production to influenza A virus infected cells. Our high-throughput screen identified a compound, ASN2, which potently inhibits influenza virus by a mechanism distinct from existing influenza antiviral drugs. This is the first report of an antiviral compound capable of inhibiting influenza virus polymerase function while simultaneously activating the innate immune system.

## Results

### Identification of ASN2

**Figure 1:**
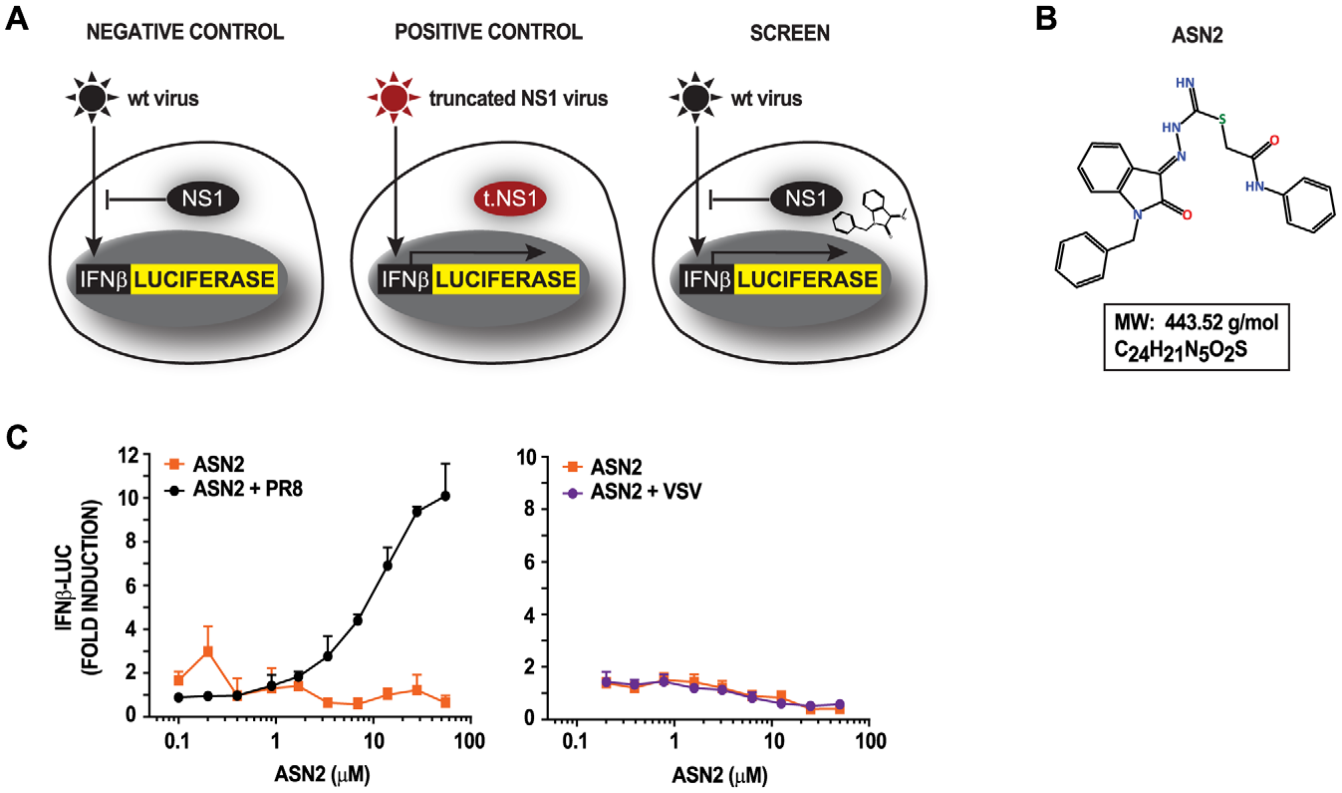
Concept for high-throughput compound screen and identification of ASN2. (A) MDCK cells stably expressing IFNβ-luciferase reporter are not responsive to wild type (wt) influenza A virus infection due to the presence of a fully functional NS1 protein (left panel). Infection with a mutant virus expressing a truncated NS1 protein (rPR8 NS1-113), which is unable to antagonize the IFNβ production pathway, can induce the IFNβ-luciferase reporter (middle panel). The HTS assay consisted of infecting the reporter cells with wt influenza A virus in the presence of small molecular weight compounds. The aim was to identify compounds that are able to restore IFNβ in the presence of wt influenza A virus (right panel). (B) Chemical structure of ASN2 with its molecular weight (MW) and chemical formula. (C) Reporter assays of MDCK IFNβ-luciferase cells treated with increasing concentrations of ASN2 for 2 hours prior to mock infection or infection with influenza A/PR/8/34 virus and VSV-GFP. Luciferase activity was assayed 18 hours post infection. Curves represent the mean of triplicate values ± standard deviation.

For screening small molecular weight compounds in a high-throughput format, it was essential to develop a primary screen assay that was simple, fast, and robust. For this purpose, we took advantage of an MDCK stable reporter cell line (MDCK IFNβ-luciferase) [23] which provides an easy measurement of IFN induction. When these cells are infected with wild type influenza virus, expression of the reporter gene is not activated due to the presence of the virally encoded NS1 protein, which blocks virus-induced activation of the IFNβ-promoter (Fig. 1A). However, infection with a mutant influenza virus containing a non-functional NS1 protein (rPR8 NS1-113 [21]) results in robust activation of the reporter. The screen assay consisted of infection of the MDCK IFNβ-luciferase reporter cells with wild type influenza A/PR/8/34 virus in the presence of the library compounds, with the aim of identifying those that restore the IFNβ response.

The primary screen assay was initially optimized in a 96-well format and further miniaturized into 384-well format. We verified its suitability for use in a high-throughput screen by the following parameters: a Z′ factor of 0.5 [24], coefficient of variation (CV) of 16.64±0.07, signal-to-background ratio (S/B) of 84.69±0.45, and signal-to-noise ratio (S/N) of 5.94±0.02. A library of 84,551 structurally diverse compounds was screened in duplicate at the National Screening Laboratory for the Regional Centers of Excellence in Biodefense and Emerging Infectious Diseases (NSRB) (Harvard Medical School) and hits were identified based on an increase in luciferase signal. The results from the primary screen were standardized by calculating a Z-Score for each compound (Fig. S1). Hit compounds were selected based on minimum Z-Scores of 3 and 2.5 from duplicate samples, which resulted in the identification of 264 primary hits (0.3%).

Secondary screen assays were performed in order to confirm the hits identified in the primary screen. Every hit compound was tested in a 10-point dose-response curve both in the presence and absence of influenza virus infection in order to distinguish between those compounds that induce IFNβ independently of a virus stimulus versus those that require virus infection. This was in contrast to the primary screen, in which each compound was tested at a single concentration (350 fold dilution of the stock concentration, see methods), and only in the presence of influenza virus infection. A compound was confirmed as a hit if it displayed a dose-dependent effect with a minimum 5 fold induction either in the presence or absence of influenza virus infection. This process resulted in the selection of 27 compounds, of which 25 induced IFNβ induction independently of virus infection and two induced IFNβ induction only in the presence of virus infection. Since the latter two compounds are likely to induce a specific antiviral immune response only in infected cells, thus limiting the undesirable side effects associated with IFN therapy, these were pursued for further characterization. We selected ASN2 (Fig. 1B) as our lead compound since this compound produced a more robust IFN response.

To explore the requirement for an influenza virus-specific stimulus, we tested the ability of ASN2 to induce IFNβ expression in the presence of an influenza virus and a non-influenza virus, vesicular stomatitis virus (VSV). ASN2 was able to induce IFNβ in the presence of influenza A/PR/8/34 virus but not VSV (Fig. 1C), suggesting that this compound requires an influenza virus-specific stimulus in order to induce IFNβ.

### ASN2 induces an antiviral response during influenza A virus infection

**Figure 2:**
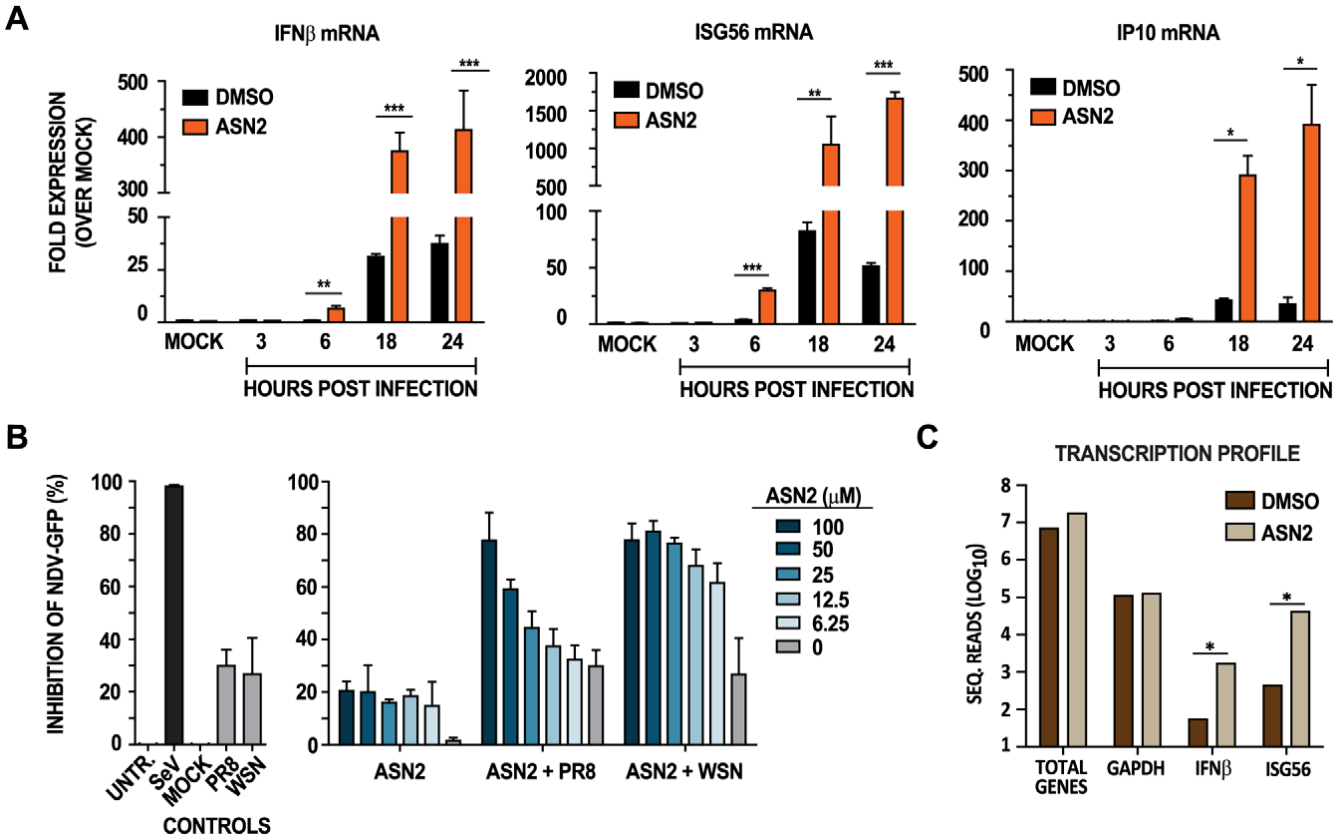
ASN2 induces an antiviral response during influenza A virus infection. (A) qRT-PCR analysis of IFNβ, ISG56 and IP10 transcripts in A549 cells infected with influenza A/WSN/33 virus (MOI = 1) and treated with ASN2 (50 µM) for 24 hours. Values were normalized to α-tubulin for each sample and are represented as fold induction over uninfected DMSO-treated sample (MOCK). Error bars reflect standard deviation of fold change. p<0.05, **p<0.005, ***p<0.0005 (B) Antiviral bioassay analysis of A549 cells infected with influenza A virus and treated with ASN2 for 18 hours. Supernatants from these cells were collected and UV-inactivated prior to overlaying them onto freshly plated VERO cells for a 24 hour incubation period prior to infection with NDV-GFP. Graphs represent percent inhibition of NDV-GFP induced by the supernatants collected from the indicated treatments. Percentage inhibition induced by supernatants from Sendai virus (SeV) infection was set to 100%. Bars represent mean of triplicate values ± standard deviation. (C) Deep sequencing analysis of mRNA from A549 cells infected with A/WSN/33 (MOI = 1) and treated with DMSO or ASN2 (50 µM) for 24 hours. The total number of reads obtained for the indicated genes for DMSO and ASN2 treatments are shown. *p<0.05.

To verify that the IFNβ-luciferase reporter induction observed in the primary and secondary screens correlates with induction of IFNβ mRNA and associated IFN-stimulated genes (ISG) in human cells, we infected human lung carcinoma (A549) cells with influenza A/WSN/33 virus, treated them with either ASN2 or DMSO, and examined expression of IFN-related genes by qRT-PCR. IFNβ, ISG56 (IFIT1), and IP10 (CXCL10) mRNAs were significantly induced in the infected sample treated with ASN2, and this induction increased over time (Fig. 2A). Other genes tested by qRT-PCR showed similar patterns of induction in the presence of ASN2, including IFNλ-1, -2, -3, CCL5, IRF7, ISG54, MxA, PKR, and STAT1 (data not shown).

To explore whether the cytokines released during ASN2 treatment of influenza virus infected cells are able to induce a protective antiviral response, we performed an antiviral bioassay. A549 cells were infected with influenza virus and treated with ASN2 or DMSO. Infection with Sendai virus (SeV) Cantell was used as a positive control for interferon induction [25]. Supernatants were collected at 24 hours, UV-inactivated and then transferred to naïve VERO cells (which are defective for production of interferon but sensitive to the action of exogenous interferon [26], [27], [28]), for 24 hours prior to infection with Newcastle disease virus expressing a GFP reporter (NDV-GFP). If the supernatants contain cytokines capable of inducing an antiviral state, subsequent infection with NDV-GFP would be restricted; otherwise, growth of NDV-GFP would be expected to proceed uninhibited. Supernatants from influenza virus infected cells treated with ASN2 were capable of restricting NDV-GFP growth in VERO cells, and this effect was directly proportional to the dose of ASN2 (Fig. 2B). As expected, supernatants from influenza virus infected cells treated with DMSO did not restrict growth of NDV-GFP as wild-type influenza virus infection does not induce significant levels of IFNβ. Notably, supernatants from uninfected ASN2-treated cells only showed minimal ability to restrict the growth of NDV-GFP. As this assay measures general antiviral cytokine responses, not only type I IFN, it is possible that ASN2 is capable of inducing some non-IFN cytokines that account for the observed 20% inhibition of NDV-GFP. Overall these data indicate that only cells both infected with influenza virus and treated with ASN2 are capable of releasing cytokines that induce a robust antiviral state in VERO cells.

To further understand the transcriptional profile of genes affected by ASN2 treatment, we performed deep sequencing of total mRNA from A549 cells infected with influenza A/WSN/33 virus and treated with either ASN2 or DMSO. The total number of sequence reads for each gene was normalized to that of GAPDH for each sample, since this gene was unaffected by ASN2 and was one of the most abundant. We compared a total of 15,154 genes and 34 of them were induced at least 100 fold in the ASN2 sample over the DMSO control (Table S1). Amongst these induced genes are several known ISGs (Fig. 2C), and notably, several have been described to participate in the innate immune response to viral pathogens (e.g. Mx, OAS, IFIH1 (MDA5), BST2 (tetherin), DDX58 (RIGI)). This analysis further demonstrated the ability of ASN2 to induce the expression of genes that are associated with the innate immune response.

To address whether viral replication was required for ASN2-mediated induction of IFNβ, an infection with UV-inactivated influenza virus was performed. Indeed, ASN2 was incapable of inducing IFNβ and ISG56 mRNA in the presence of UV-inactivated influenza A virus, contrary to its effect in the presence of a replication-competent virus (Fig. S2). This indicated a necessity for virus replication in the IFNβ-inducing activity of ASN2.

### Antiviral activity of ASN2

**Figure 3:**
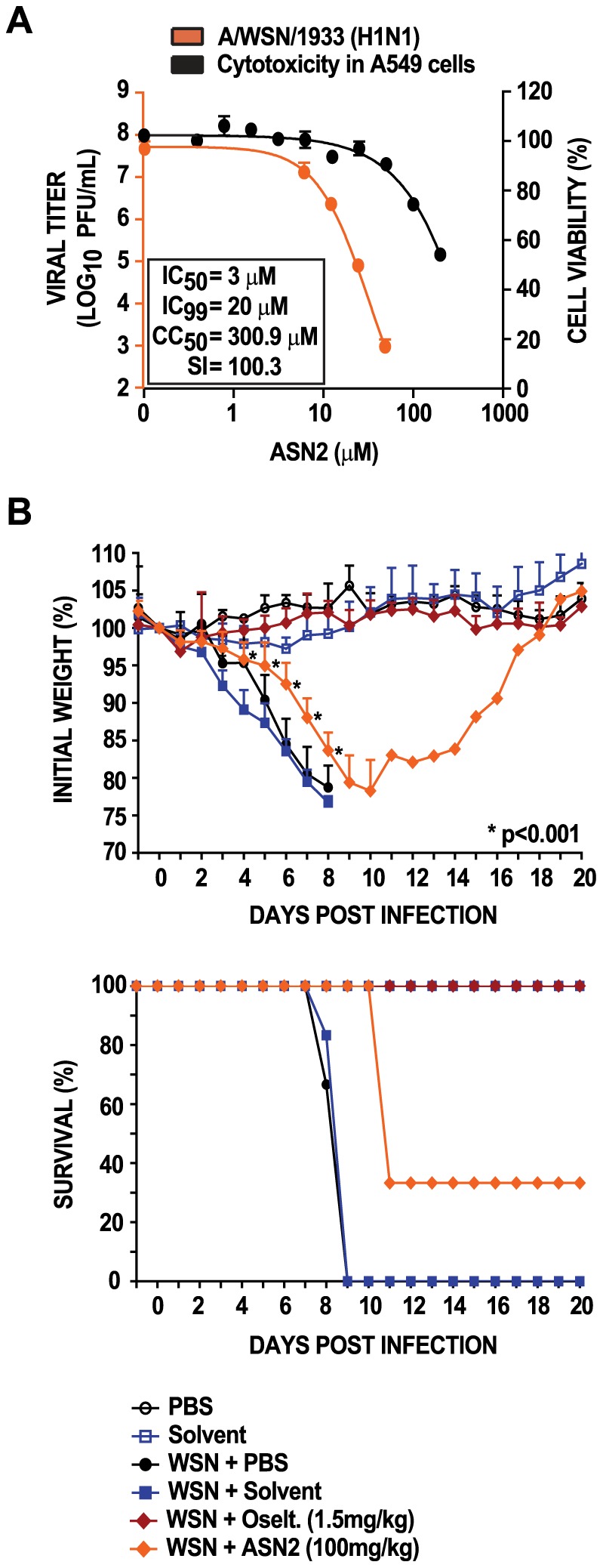
ASN2 inhibits the replication of influenza A virus and displays *in vivo* antiviral activity. (A) Virus titers from A549 cells infected with influenza A/WSN/33 virus (MOI = 0.01) and treated with increasing concentrations of ASN2 for 48 hours (orange curve). Cell viability analysis of A549 cells treated with increasing concentrations of ASN2 for 48 hours (black curve). Curves represent means of triplicate values ± standard deviation. (B) Bodyweight and survival curves of BALB/c mice (groups of 9) infected with influenza A/WSN/33 virus (5LD_50_) and treated with 100 mg/kg of ASN2 every 8 hours for 8 days. Compound was delivered intraperitoneally beginning 8 hr prior to infection. Three mice from each group were sacrificed on days 3 and 8 post infection to determine viral lung titers (data not shown). Curves represent means ± standard deviation, *p<0.001. Mice that fell below 75% of their initial weight were sacrificed in accordance with our animal protocol.

To assess its potential to inhibit virus replication, ASN2 was tested at non-cytotoxic concentrations in a viral replication assay. A549 cells were infected with influenza A/WSN/33 virus (MOI = 0.01) and treated with ASN2 at a range of concentrations for 48 hours. A reduction in viral titers of 4.5 logs was detected in the presence of 50 µM ASN2, and the IC_50_ (concentration of 50% inhibition) was determined to be 3 µM (Fig. 3A). The CC_50_ (concentration of 50% cytotoxicity) for ASN2 is 300.9 µM, resulting in a selective index (SI = CC_50_/IC_50_) of 100.3. Several strains of influenza A and B viruses as well as non-influenza viruses (VSV and Sindbis virus) were tested against ASN2. Interestingly, ASN2 was significantly more potent against influenza A viruses, including a number of strains of H1N1, H3N2, and H5N1 subtypes, and it inhibited the reconstructed virus from the 1918 pandemic very efficiently, reducing viral titers by up to 6.2 logs (Table 1). We note that for non-influenza A viruses, ASN2 does display some antiviral activity but that it is far more effective against influenza A viruses. We hypothesize that this non-specific antiviral effect may be related to the minor induction of antiviral cytokines by ASN2 observed in the antiviral bioassay (Fig. 2B).

**Table 1:** Viruses tested against ASN2.

Virus Strain	IC_50_ (µM)	IC_99_ (µM)	Selective Index (CC_50_/IC_50_)	Inhibition at 50 µM (log_10_)
***Influenza Viruses***				
**rA/Brevig Mission/1/1918 (H1N1)** *	3	13.5	100.3	6.2
**A/WSN/1933 (H1N1)**	3	20	100.3	4.6
**rA/Puerto Rico/8/1934 (H1N1)**	4	24	75.3	2.4
**A/Hong Kong/1/1968 (H3N2)**	3	6	100.3	4.4
**A/Udorn/307/1972 (H1N1)**	3.5	14	86	4.7
**A/USSR/90/1977 (H1N1)**	4.2	13	71.6	3.2
**A/swine/Texas/4199-2/1998 (H3N2)**	3	12.5	100.3	4.2
**rA/Vietnam/1203/2004 (H5N1)** **	4	12	75.3	4.6
**A/California/04/2009 (H1N1)**	3	40	100.3	2.2
**B/Yamagata/16/1988**	14	N/A	21.5	0.3
***Non-Influenza Viruses***				
**Vesicular Stomatitis Virus**	5	52	60.2	1.6
**Sindbis Virus** ***	48	>50	6.5	0.2

*: HA segment from A/South Carolina/1/1918.

**: Low pathogenicity mutant (deleted polybasic cleavage site in HA).

***: Experiment done in VERO cells.

To evaluate the activity and potency of ASN2 *in vivo*, BALB/c mice were treated with ASN2 (100 mg/kg/day) every 8 hours for eight days. The first dose was given 8 hours prior to infection with 5MLD_50_ of influenza A/WSN/33 virus. PBS-treated and solvent-treated infected animals succumbed to infection at 9 days post-infection (0% survival), while the uninfected group and oseltamivir-treated group (1.5 mg/kg/day) displayed 100% survival (Fig. 3B). The ASN2-treated animals exhibited significantly less weight loss compared to the PBS-treated and solvent-treated infected animals and this resulted in a delayed time-to-death with 33% surviving up to 20 days post infection. Mice from each group were euthanized on days 3 and 8 post infection and lungs were harvested to determine viral titers. Except for the oseltamivir-treated group, no significant differences in lung titers were observed (data not shown). The limited performance of ASN2 *in vivo* is perhaps explained by metabolic instability. An *in vitro* mouse liver microsome assay was used to predict the metabolic stability of ASN2 and the results showed a high intrinsic hepatic clearance (CL_int_) of 224 µL/min/mg protein (normal levels being 8.8–48 µL/min/mg protein), and a very short half-life (t_1/2_) of 6.18 min. Collectively, these results show that ASN2 partially protects mice from lethal influenza A virus infection, and suggest that the pharmacokinetic properties of ASN2 could be optimized to further improve *in vivo* efficacy.

### ASN2 targets influenza A virus polymerase function

**Figure 4:**
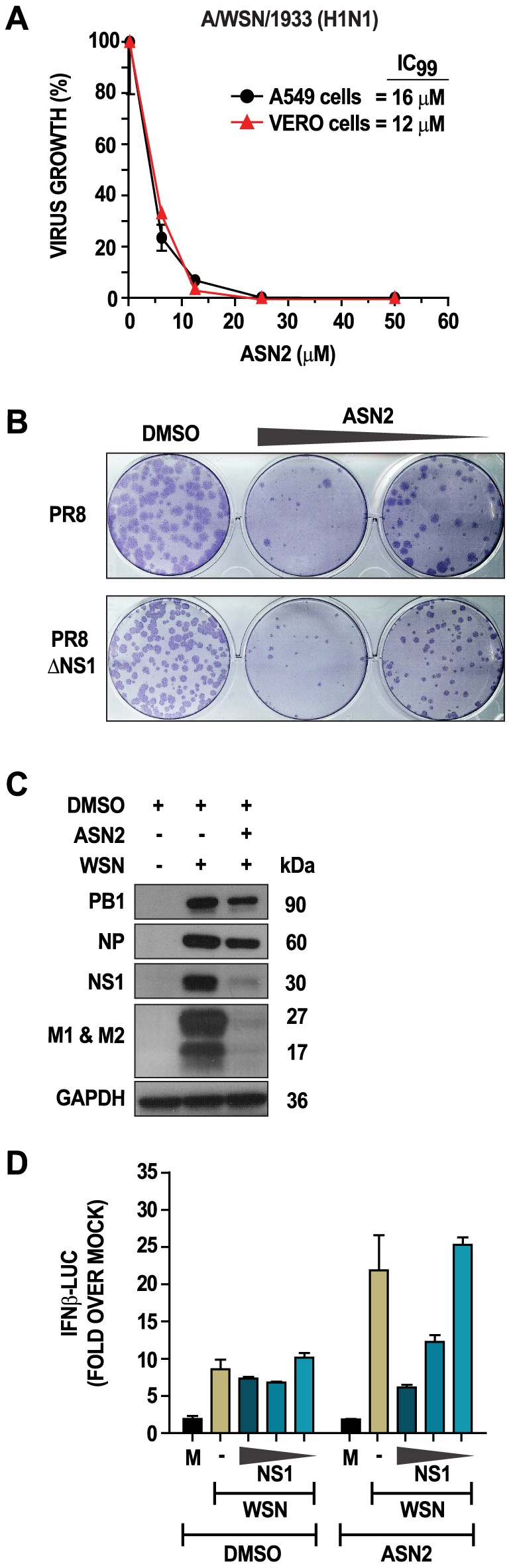
Antiviral activity of ASN2 is independent of interferon production and NS1. (A) Virus titers from A549 and VERO cells infected with influenza A/WSN/33 virus (MOI = 0.01) and treated with increasing concentrations of ASN2 (6.25–50 µM) for 48 hours. (B) Plaque-reduction analysis of VERO cells infected with the indicated viruses in the presence of either DMSO or ASN2 (50 or 25 µM). Plaques were immunostained using an NP antibody. (C) Western blot analysis of cellular extracts from A549 cells infected with influenza A/WSN/33 virus (MOI = 1) and treated with ASN2 (50 µM) for 24 hours. Specific antibodies were used for each of the indicated proteins. (D) Reporter assay analysis of A549 cells transfected with IFNβ-luciferase and pRL-TK reporters, empty plasmid (-) or decreasing concentrations of NS1 (325, 32.5, and 3.25 ng) for 24 hours prior to infection with influenza A/WSN/33 virus (MOI = 1). Cells were treated with either ASN2 (50 µM) or DMSO and luciferase activity was determined 24 hours post infection. Values were normalized to *Renilla* luciferase activity for each sample and are represented as fold induction over uninfected DMSO-treated sample (mock). Bars represent means of triplicate values ± standard deviation.

To determine the contribution of IFN to the antiviral activity of ASN2, we performed virus inhibition assays in A549 cells and VERO cells simultaneously. Cells were infected with influenza A/WSN/33 virus (MOI = 0.01) and then treated with increasing concentrations of ASN2 for 48 hours prior to measuring virus titers in the supernatants. Surprisingly, antiviral activity was still observed in VERO cells, which are known to be defective for the production of type I IFN, with negligible differences in their IC_50_ and IC_99_ concentrations as compared to A549 cells (Fig. 4A). The same results were obtained when using an even lower multiplicity (MOI = 0.0001) in A549 and VERO cells, which should have allowed for any IFN-mediated inhibition to be observed (data not shown). Moreover, ASN2 also retained full activity in cells deficient in IRF9, STAT1, or STAT2 (data not shown). These results suggest that the antiviral activity of ASN2 in tissue culture is independent of IFN activity.

Due to the role of the influenza A virus NS1 protein as a potent IFN antagonist and facilitator of viral replication, we investigated the involvement of this protein in the antiviral activity of ASN2. VERO cells were infected with wild type influenza A/PR/8/34 virus or a virus lacking NS1 (PR8ΔNS1) in the presence of DMSO or ASN2. ASN2 treatment significantly reduced the number of plaques relative to DMSO treatment for both wild type and ΔNS1 viruses (Fig. 4B), suggesting that NS1 is not a direct target of ASN2.

We next explored viral protein expression in influenza virus infected cells under conditions where IFNβ induction is observed in the presence of ASN2. A549 cells were infected with influenza A/WSN/33 virus (MOI = 1) and treated with 50 µM ASN2 for 24 hours. Western blot analysis revealed decreased expression of all viral proteins examined but this decrease was much more apparent for the NS1, M1 and M2 proteins (Fig. 4C). To associate this loss of NS1 expression with the induction of IFNβ observed during ASN2 treatment, NS1 was expressed from plasmid in A549 cells infected with influenza virus and treated with ASN2. Infected cells transfected with empty plasmid displayed induction of IFNβ in the presence of ASN2 and not DMSO, as seen previously (Fig. 4D). On the contrary, IFNβ induction was not observed in ASN2-treated infected cells overexpressing NS1. Furthermore, dilution of the NS1 plasmid resulted in dose-dependent restoration of IFNβ induction in these cells. These findings suggest that the loss of NS1 expression, which results from ASN2-mediated virus inhibition, is responsible for the observed induction of IFNβ.

**Figure 5:**
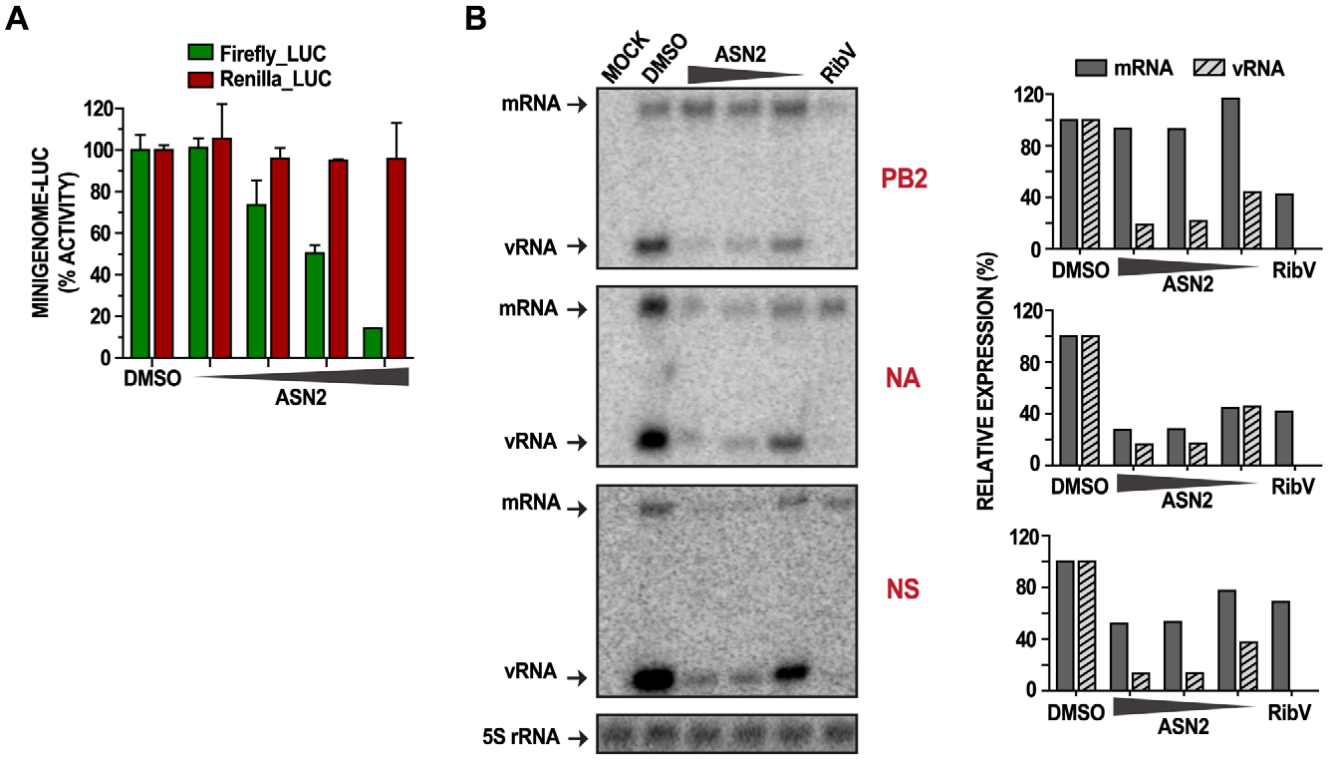
ASN2 inhibits viral RNA synthesis. (A) Influenza A virus mini-genome activity in A549 cells transfected with the influenza virus firefly luciferase mini-genome reporter, pRL-TK control reporter, PB1, PB2, PA, and NP protein expressing plasmids. Treatment with DMSO or increasing concentrations of ASN2 (6.25–50 µM) was performed during transfection. Luciferase activity was assayed 24 hours post transfection. Firefly and Renilla luciferase values are represented as % activity from the mock (minigenome without NP expression) relative to the DMSO control. Error bars reflect the standard deviation of % activity. (B) Primer extension analysis of influenza virus mRNA and vRNA from A549 cells infected with influenza A/WSN/33 virus (MOI = 5) and treated with DMSO or decreasing concentrations of ASN2 (50–12.5 µM) for 6 hours. PB2, NA, and NS segments were analyzed. Ribavirin (100 µM) was used as a positive control and 5S rRNA was used as a loading control. Quantification of mRNA and vRNA expression, normalized to 5S rRNA, is shown on the right.

Given that ASN2 treatment resulted in decreased viral protein expression, the effect of ASN2 on replication and/or transcription of viral RNA was investigated. An influenza virus mini-genome assay, which measures the activity of the viral polymerase complex in the context of a reconstituted viral ribonucleoprotein, was performed in the presence or absence of ASN2. A549 cells were co-transfected with expression plasmids for the influenza A/WSN/33 virus polymerase proteins (PB1, PB2, and PA), the nucleoprotein (NP), an influenza virus-specific luciferase reporter plasmid [29], and a transfection control plasmid. ASN2 or DMSO was added and the transfection allowed to proceed for 24 hours prior to examining luciferase activity. ASN2 strongly inhibited the influenza virus mini-genome reporter in a dose-dependent manner without affecting expression of the control reporter, suggesting that the function of the influenza virus polymerase was affected by ASN2 (Fig. 5A).

These findings were complemented by performing primer extension assays to analyze the effect of ASN2 on different influenza virus RNA species. A549 cells were infected with influenza A/WSN/33 virus (MOI = 5) and treated with ASN2 for 6 hours prior to harvesting total RNA. Production of mRNA and vRNA from three representative segments of the influenza genome, PB2, NA, and NS, was analyzed. Treatment with ribavirin was included as a control. For all three segments, vRNA synthesis was inhibited by ASN2 in a dose-dependent manner, whereas mRNA synthesis was decreased in the NA and NS segments but not in the PB2 segment (Fig. 5B). In contrast, the ribavirin control inhibited all three segments to an equal extent. From these results we propose that ASN2 inhibits polymerase function in a manner that inhibits vRNA synthesis from all segments and that it also perturbs mRNA production but possibly in a segment-specific manner.

The effects of ASN2 on viral mRNA synthesis were verified by analyzing the transcriptional profile of viral mRNA obtained by deep sequencing of infected cells treated with ASN2 or DMSO, as described previously. A striking segment-dependent effect on the production of viral mRNA in the ASN2-treated sample was observed (Fig. S3). Notably, transcription from the smaller segments, M and NS, was inhibited to the greatest extent, which correlated with the results from the primer extension and western blot assays showing that mRNA and protein levels from smaller segments were more strongly affected by ASN2 treatment than those from larger ones. An apparent increase in mRNA expression was seen with the larger segments (PB2, PB1, PA) in the presence of ASN2 (Fig. S3).

**Figure 6:**
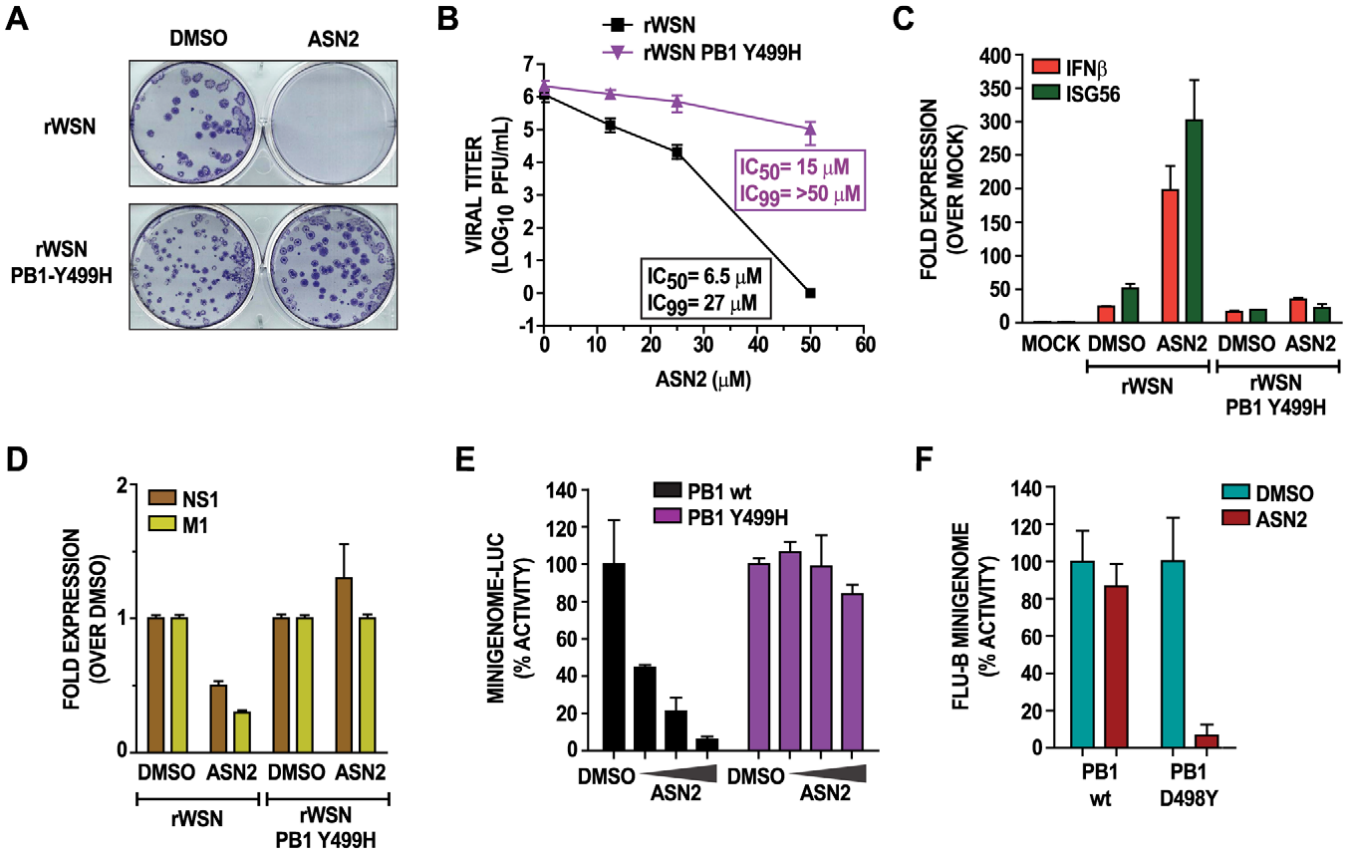
A Y499H mutation in the PB1 protein confers resistance to ASN2. (A) Plaque-reduction analysis of MDCK cells infected with the indicated viruses in the presence of either DMSO or ASN2 (50 µM). Plaques were stained with an anti-NP antibody. (B) Virus titers from MDCK cells infected with the indicated viruses and treated with increasing concentrations of ASN2 for 48 hours. Curves represent means of triplicate values ± standard deviation. (C–D) qRT-PCR analysis of IFNβ, ISG56, NS1 and M1 mRNAs from A549 cells infected with the indicated viruses (MOI = 1) and treated with ASN2 (50 µM) for 24 hours. Values in (C) were normalized to α-tubulin for each sample and are represented as fold induction over uninfected DMSO-treated sample. Values in (D) are represented as fold induction over the infected DMSO-treated sample. Error bars reflect standard deviation of fold change. (E) Influenza A virus mini-genome activity in 293T cells transfected with the influenza A virus mini-genome reporter, pRL-TK control reporter, wtPB1 or PB1-Y499H, PB2, PA, and NP protein expressing plasmids. Treatment with DMSO or increasing concentrations of ASN2 (12.5–50 µM) was performed during transfection. Luciferase activity was assayed 24 hours post transfection. Values were normalized to *Renilla* luciferase activity for each sample and are represented as % activity relative to the control (minigenome without PB1 expression). Error bars reflect the standard deviation of % activity. (F) Influenza B virus mini-genome activity in A549 cells transfected with the influenza B virus mini-genome CAT reporter, pRL-TK control reporter, wtPB1 or PB1-D498Y, PB2, PA, and NP protein expressing plasmids. Cells were treated with DMSO or 50 µM ASN2. CAT and luciferase activities were measured 24 hrs post transfection and normalized % mini-genome activity for the wt or mutant PB1 is shown relative to the DMSO control in each case.

To determine if the ASN2 target is a viral protein, we attempted to select for an ASN2 escape mutant influenza virus. A resistant virus was isolated in four passages and complete sequencing of the viral genome revealed a single amino acid change from a tyrosine (Y) to a histidine (H) at position 499 of the PB1 protein (PB1-Y499H). To confirm that the PB1-Y499H mutation indeed conferred resistance to ASN2, we engineered this mutation into a recombinant influenza A/WSN/33 virus (rWSN PB1-Y499H) using reverse genetics [30]. The PB1-Y499H mutation did not interfere with the normal growth properties of the virus (data not shown) and unlike the wild type virus, rWSN PB1-Y499H was resistant to ASN2 treatment (Fig. 6A–B).. Furthermore, the expression profiles of IFNβ, ISG56, M1, and NS1 mRNA were unaffected by ASN2 treatment of cells infected with the rWSN PB1-Y499H virus, whereas an induction of IFNβ and ISG56, and inhibition of M1 and NS1 was observed in the presence rWSN infection (Fig. 6C–D).

To verify that the PB1-Y499H mutation conferred resistance to ASN2 by restoring normal viral RNA synthesis, we performed an influenza virus mini-genome assay using vRNPs reconstituted with either wild type PB1 or PB1-Y499H. ASN2 inhibited polymerase function in the mini-genome assay using the wild type PB1, but not the PB1-Y499H mutant protein (Fig. 6E). Collectively, these findings confirmed that ASN2 resistance was conferred by a single amino acid mutation in the PB1 protein and strongly suggested that the viral target of ASN2 was the influenza A virus PB1 protein.

We examined the conservation of residue 499 among several human influenza A viruses using the influenza virus research database (IRD) [31]. Over 6,000 viruses from three different influenza A virus subtypes were analyzed, and the majority either contained a serine (S) or a tyrosine (Y) at position 499 of the PB1 protein, with exception of one A/H3N2 virus which contained a phenylalanine (F) (Table S2). This indicates that residue 499 of PB1 is highly conserved among influenza A viruses, which correlates with the ability of ASN2 to inhibit all influenza A viruses examined in this study. The corresponding amino acid position in the PB1 protein of influenza B viruses is an aspartic acid (D498), which may explain the relatively inefficient ASN2 antiviral activity observed with influenza B virus (Table 1). Surprisingly, it was found that mutation of residue D498 to Y rendered the influenza B virus polymerase sensitive to inhibition by ASN2, as seen in a mini-genome assay (Fig. 6F), although this mutant polymerase was less active than that containing the wild-type PB1.

Examination of this amino acid position in polymerases of all segmented negative strand RNA viruses shows that it is in close proximity (5 residues downstream) to Motif E, which is a highly conserved region [32]. Nevertheless, the residue at this position differs between viruses, with no other virus possessing a tyrosine or a serine as do influenza A viruses. Again, this correlates with our finding that ASN2 is more specific for influenza A viruses.

## Discussion

We developed a cell-based high-throughput screen assay that identifies small molecular weight compounds which are able to restore the endogenous antiviral response to influenza virus infected cells. In this process, we identified two groups of IFNβ-inducing compounds. The first group includes 25 compounds that induce IFNβ independently of a virus stimulus while the second group consists of two compounds that induce IFNβ only in the presence of virus. From the latter group, ASN2 was chosen as a lead compound because of its superior ability to induce the expression of type I IFN and ISGs, and thereby establish an antiviral state; a desirable feature of antiviral drugs directed against viruses that are sensitive to the effects of IFN. This response is very specific for influenza virus and requires virus replication. Transcriptional profiling of influenza virus infected cells treated with ASN2 confirmed the ability of this compound to induce a myriad of genes, many of which are known ISGs with potent antiviral function. However, a more in-depth analysis of these data will be necessary to fully understand the spectrum of cellular genes induced by ASN2 that may contribute to its antiviral activity. In addition, we confirmed the biological activity of the ASN2-induced cytokines in an antiviral bioassay, indicating that this response is capable of protecting neighboring, uninfected cells.

ASN2 has potent antiviral activity against multiple subtypes of influenza A virus, including several pandemic strains that have been associated with severe human disease. This antiviral activity mediates partial protection of mice from a lethal dose of influenza A virus, a feature that can probably be improved further with medicinal chemistry optimization of ASN2 to provide optimal pharmacokinetic properties. Given the strong IFN-inducing ability of ASN2 we were particularly surprised to find that the compound retained its antiviral activity in Vero cells, indicating that its antiviral mechanism is independent of type I IFN. Thus it appeared that the activation of an innate immune response was secondary to a more direct antiviral function. The ability of ASN2 to inhibit influenza virus mini-genome activity suggested that viral polymerase function may be the target of the compound. Generation of an ASN2 resistant virus revealed the importance of a tyrosine residue at position 499 of the PB1 protein for ASN2 antiviral activity.

Position 499 of the PB1 protein is occupied by either a tyrosine or a serine in virtually all influenza A virus strains, but not in the polymerases of other segmented negative-stranded viruses, including the PB1 proteins of influenza B and C viruses, which have an aspartic acid and a glycine, respectively in this position. This variability explains the observed spectrum of ASN2 antiviral activity and we demonstrated that the influenza B virus polymerase, which is naturally insensitive to ASN2, can be made to be ASN2-sensitive by simple substitution of a tyrosine for the wild-type aspartic acid. It is tempting to speculate that the requirement for a tyrosine or serine residue is an indication that these residues are targets for phosphorylation and that ASN2 somehow interferes with this modification, although the presence of histidine in the resistant mutant may not support this theory. Of note, PB1 is reported to be phosphorylated by protein kinase C, but the target residue has not been identified [33]. Nevertheless, our data definitely suggest a functional role for this particular region of the PB1 protein, which has not been elucidated to date. The rapid generation of resistance is a strong indication that PB1 is the direct target of ASN2 (as opposed to a cellular protein) but this will have to be formally proven with *in vitro* binding assays. Unfortunately this region of PB1 is not covered by the available crystal structures of PB1 so *in silico* modeling attempts are not possible at this time.

Despite the inhibitory effects of ASN2 on polymerase function in the mini-genome assay, when viral gene expression was analyzed at the level of both mRNA and protein it was clear that not all genes are equally affected. In particular, expression of genes encoded by the smallest genome segments, M and NS, are more severely inhibited. The suppression of NS1 protein expression immediately led us to question whether this was the cause of ASN2-mediated IFN induction and supplementation with plasmid-expressed NS1 showed that this is indeed the case. Thus, the induction of IFN observed in ASN2-treated influenza virus infected cells appears to be due to loss of NS1 expression, which is secondary to a specific inhibitory effect of ASN2 on viral polymerase function. Obviously it will be important to assess the consequences of reduced M1, M2, and NEP expression as well and determine how they may contribute to the antiviral activity of ASN2. In particular, NEP has been shown to be a key regulator of viral transcription and replication, and lack of NEP may facilitate the antiviral actions of ASN2, possibly through its role in the production of svRNAs [34], [35].

To our knowledge, differential viral gene expression based on segment length has not been described for influenza virus. Rather, it has been reported that some genes are preferentially expressed early (NP and NS1) and others late in infection (M1) [36], [37], however this pattern clearly does not correlate with what we observed in the presence of ASN2 where there was preferential downregulation of mRNAs expressed from the smaller segments (M1, M2, NS1, NEP). This effect seems to be specific for transcription as no segment-dependent inhibition of vRNA synthesis was observed. Although the M and NS segments are coincidentally also the ones that produce spliced products, we do not think that ASN2 is specifically affecting splicing as we observe similar decreases in proteins expressed from both spliced (M2, NEP) and unspliced (M1, NS1) transcripts. In the absence of a better understanding of viral transcription regulation it is difficult to interpret these findings but it could point to a role for PB1 (or one of the smaller viral gene products) in regulating mRNA synthesis in a segment-dependent manner. In that sense ASN2 could serve as a useful tool to investigate this phenomenon further.

It should be noted that the ability to select for an ASN2-resistant mutant in tissue culture does not necessarily impact negatively on the future of ASN2 as an antiviral drug candidate. Similar findings have been reported with oseltamivir which is a very successful influenza virus drug [38], [39]. Furthermore, it is likely that future influenza therapies will involve combinations of drugs that act via distinct mechanisms, thereby reducing the chances of drug resistance. In this respect, the identification of ASN2 as a polymerase inhibitor complements the current class of neuraminidase inhibitors. To date, there has been only one other report of an influenza antiviral compound targeting PB1 [40]. This compound differs structurally from ASN2 and also generates a different resistance mutation (H456P), but without a three dimensional structure we cannot predict whether amino acids 499 and 456 are in close proximity. The type I IFN-inducing capacity of ASN2 is a unique property for a virus-directed compound and combines both viral inhibitory activity and immune activation activity in one molecule. We believe that the ability of ASN2 to preferentially inhibit NS1 expression is the reason for its IFN induction properties and that other inhibitors of influenza virus polymerase function that act equally on all segments will not induce IFN. In short, ASN2 produces an imbalance in the synthesis of RIG-I-stimulating RNA versus NS1, whereas antiviral compounds affecting all segments suppress both the production of RIG-I stimulus and NS1. In support, we have shown that A3, a broad-spectrum antiviral compound that depletes pyrimidine pools and prevents all viral RNA synthesis [41], does not induce IFN (Fig. S4). Although not essential for antiviral action in tissue culture, IFN induction may prove to be beneficial in an *in vivo* setting, possibly facilitating activation of the adaptive immune response and viral clearance, which will be explored further using IFNAR^−/−^ mice. The targeted production of IFN (i.e. only occurring in influenza virus infected cells) also provides distinct advantages over exogenous IFN therapies which are often associated with unpleasant side effects.

## Materials and Methods

### Cell culture and reagents

Madin-Darby canine kidney (MDCK) epithelial cells, human alveolar epithelial (A549) cells, human embryonic kidney 293T (293T) cells, and African green monkey kidney cells (VERO) cells, were obtained from the American Type Culture Collection (ATCC, Manassas, VA). MDCK stably expressing the IFNβ-luciferase reporter (MDCK IFNβ-luciferase) [23] were kindly provided by Adolfo Garcia-Sastre (Mount Sinai School of Medicine, New York, NY). MDCK, A549, 293T, and VERO cells were cultured in Dulbecco's modified Eagle's medium (DMEM) (Gibco, Carlsbad, CA) supplemented with 10% fetal bovine serum (FBS) (HyClone, South Logan, UT), and 1% penicillin-streptomycin (P/S) (Gibco). MDCK IFNβ-luciferase cells were cultured in DMEM supplemented with 10% FBS, 1% P/S, 0.5 mg/mL Hygromycin B (Invitrogen, Carlsbad, CA), and 2 mg/mL Geneticin (Invitrogen). All cells were grown at 37°C, 5% CO_2_.

Treatment of cells with human β interferon (IFNβ) (PBL Interferon Source, Piscataway, NJ) was performed as described in the text. Transfection of DNA and RNA was performed in Opti-MEM I-reduced serum medium (Opti-MEM) (Gibco) with Lipofectamine LTX (Invitrogen) in A549 cells according to manufacturer's specifications. For measurement of luciferase production in reporter assays, the Dual-Glo Luciferase Assay System (Promega, Fitchburg, WI) was used. The Bright-Glo Luciferase Assay System was used in the high-throughput primary screen and confirmation screen (Promega, Fitchburg, WI).

### Expression plasmids and cloning

The pRL-TK (Promega) reporter contains a *Renilla* luciferase gene under the regulation of the herpes simplex virus thymidine kinase promoter. Mammalian expression plasmids encoding the WSN NS1 protein and the firefly luciferase reporter (pGL4.17, Promega) under the regulation of the IFNβ promoter (IFNβ-LUC), were kindly provided by Adolfo Garcia-Sastre (Mount Sinai School of Medicine, New York, NY). The influenza A virus minigenome reporter (pPolI NP_Luc) was generated as previously described [29].

The influenza virus rescue plasmid pPolI-PB1 Y499H was generated by exchanging one nucleotide in the parental plasmid pPolI-PB1 using the QuickChange site-directed mutagenesis kit (Agilent Technologies, Wilmington, DE) using specific primers (forward: 5′-CACAAGTTTTTTCTATCGTCATGGGTTTGTTGCCAATTTCAGC-3′; reverse: 5′-GCTGAAATTGGCAACAAACCCATGACGATAGAAAAAACTTGTG-3′). Presence of the mutation was confirmed by sequencing (Genewiz, South Plainfield, NJ). The mammalian expression vector pCAGGS containing a chicken β-actin promoter has been previously described [42]. The expression plasmid encoding the PB1 Y499H mutation was generated by digestion of the pPolI PB1 Y499H and pCAGGS-PB1 plasmids with MfeI and AgeI enzymes (New England Biolabs, Ipswich, MA). The digested insert containing the Y499H mutation was inserted into the digested pCAGGS-PB1 plasmid. Proper insertion and presence of mutation was confirmed by sequencing (Genewiz). The pCAGGS expression plasmid containing the D498Y mutation in the influenza B/Yamagata/88 virus PB1 was generated by site-directed mutagenesis using the following primers:

Forward: 5′-GAATTTACAAGCATGTTCTATAGATATGGATTTGTATCTAATTTTGCAA

Reverse: 5′-TTGCAAAATTAGATACAAATCCATATCTATAGAACATGCTTGTAAATTC

### Viruses

The influenza A/WSN/33 (H1N1) virus (WSN) was propagated in MDCK cells for 2 days at 37°C. Influenza A/California/04/2009 (H1N1) virus was propagated in MDCK cells for 3 days at 35°C. Influenza rA/Brevig Mission/1/1918 (H1N1) virus bearing the HA segment from A/South Carolina/1/1918 was propagated in MDCK cells for 2 days at 37°C. Experiments involving this virus were conducted under Biosafety level 3-enhanced conditions in accordance with the CDC guidelines. Influenza viruses A/Puerto Rico/8/1934 (H1N1) (rPR8), A/Hong Kong/1/1968 (H3N2), A/Udorn/307/1972 (H3N2), A/USSR/90/1977 (H1N1), A/swine/Texas/ 4199-2/1998 (H3N2), and rA/Vietnam/1203/2004 (H5N1) bearing a mutated polybasic cleavage site in the HA segment, and NDV-GFP were propagated in 10-day old embryonated chicken eggs for 2 days at 37°C. Sendai virus Cantell (SeV) was propagated in 8-day old embryonated chicken eggs for 2 days at 37°C. Influenza B/Yamagata/16/1988 virus was propagated in 8-day old embryonated chicken eggs for 3 days at 33°C. Influenza rPR8 NS1-113 virus was propagated in 7-day old embryonated chicken eggs for 2 days at 37°C [21]. The rescue of NDV-GFP was previously described [43]. All influenza viruses were titered by standard plaque assay in MDCK cells. Sindbis virus (SinV) and vesicular stomatitis virus expressing the green fluorescence protein (VSV-GFP) were kindly provided by John Hiscott (McGill University, Montreal, QC, Canada) and were grown and titered by plaque assay in VERO cells.

Recombinant influenza viruses were generated using the influenza virus rescue protocol as previously described [30]. Briefly, 293T cells were transfected with eight pPolI constructs expressing the PB1 (or PB1 Y499H), PB2, PA, NP, HA, NA, M, and NS genomic segments as well as pCAGGS expression plasmids encoding the PB1, PB2, PA, and NP proteins. Twenty-four hours post transfection, MDCK cells were co-cultured with the transfected 293Ts for an additional 24–48 hours, until cytopathic effects were observed. Newly generated viruses were collected and plaque-purified, and the presence of the mutation was confirmed by sequencing.

### Small molecular weight compounds

All compounds were provided by and screened at the National Screening Laboratory for the Regional Centers of Excellence in Biodefense and Emerging Infectious Diseases (NSRB) (Harvard Medical School, Boston, MA). The libraries screened were the following: Biomol ICCB Known Bioactives2 Library (480 compounds; BIOMOL, Plymouth Meeting, PA), NINDS custom collection 2 (1,040 compounds; MicroSource Discovery Systems, Gaylorsville, CT), Prestwick1 Collection (1,120 compounds; Prestwick Chemical, Inc., Washington, DC), ActiMol Tim Tec 1 (8,518 compounds; TimTec Inc., Newark, DE), Asinex 1 (12,378 compounds; Asinex Corp., Winston-Salem, NC), Bionet 2 (1,700 compounds; Ryan Scientific, Mount Pleasant, SC), Chembridge 3 (10,560 compounds; ChemBridge Corp., San Diego, CA), ChemDiv 2 (8,560 compounds; ChemDiv, Inc., San Diego, CA), ChemDiv 4 (1,320 compounds; ChemDiv, Inc., San Diego, CA), Enamine 2 (26,576 compounds; ENAMINE Ltd., Kiev, Ukraine), Life Chemicals 1 (3,893 compounds; Life Chemicals Inc., Burlington, ON), Maybridge 4 (4,576 compounds; Maybridge Ltd., Trevillett, England), Maybridge 5 (3,212 compounds; Maybridge Ltd., Trevillett, England), Mixed Commercial Plate 5 (268 compounds from ChemDiv and Maybridge libraries), Peakdale 2 (352 compounds; Peakdale Molecular Ltd. Chapel-en-le-Frith, High Peak, UK). The chemical libraries were stored at −20°C in desiccated storage containers. Compounds were dissolved in 100% dimethylsulfoxide (DMSO) at 2 mg/mL for the Prestwick1 library, 10 mM for the NINDS library, and 5 mg/mL for the remaining libraries. The dilution factor for the screen was 350 fold.

For secondary analyses, hit compounds were purchased directly from the vendors indicated and dissolved in 100% DMSO. The final concentration of DMSO in the culture medium did not exceed 0.5%.

### High-throughput screen assay

The screen was performed in duplicate using solid white 384-well plates. The MDCK IFNβ-luciferase cells were cultured to 90% confluency, washed with phosphate buffered saline (PBS) (Invitrogen), trypsinized with 0.05% Trypsin-EDTA (Invitrogen), and resuspended in phenol red-free DMEM growth medium (Invitrogen) supplemented with 5% FBS, and 1% P/S at a concentration of 2×10^5^ cells/mL. With the automated plate filler, 30 µL of the diluted MDCK IFNβ-luciferase cell suspension was seeded into each well of a 384-well plate (6000 cells/well), and incubated at 37°C, 5% CO_2_ for 20 hours. Compounds (100 nL) were then added using the Epson pin transfer robot (Epson America, Long Beach, CA), and incubated at 37°C, 5% CO_2_ for 2 hours. Cells were then infected with rPR8 (MOI = 10) by adding 5 µL of the virus inoculum (medium+1 µg/mL TPCK) directly into the medium. Two columns on the right side of each plate were reserved for controls and did not contain compounds. The second to last column was infected with only rPR8 virus (negative control) and the last column was infected with only rPR8 NS1-113 virus (positive control). Infection proceeded at 37°C, 5% CO_2_ for 18 hours prior to measuring luciferase production. 35 µL of the Bright-Glo reagent (diluted 3 fold with PBS as described above) was added to each well and the luminescence signal was measured using the EnVision plate reader. Calculation of the Z-Score and selection of hits are described in the ‘data analysis’ section.

### Data analysis

To evaluate the robustness of our assays we calculated a Z′-Factor [24]. The calculations were done according to the following formula: Z′ = 1−((3δ_pos_+3δ_neg_)/(μ_pos_−μ_neg_)), where μ_pos_ is the mean signal for the positive control (rPR8 NS1-113 infection), μ_neg_ is the mean signal for the negative control (rPR8 infection), δ_pos_ is the standard deviation of the positive control, and δ_neg_ is the standard deviation for the negative control. A reproducible Z′-Factor>0.5 is considered optimal for HTS. The percent coefficient of variation: %CV = δ_pos_/μ_pos_*100, the signal-to-background ratio: S/B = μ_pos_/μ_neg_, and the signal-to-noise ratio: S/N = (μ_pos_−μ_neg_)/((δ_pos_)∧2+(δ_neg_)∧2)∧1/2 were also calculated.

Analysis of the results obtained from the compound screen was done in Microsoft Office Excel using a Z-Score calculation to standardize each compound signal. The Z-Score formula indicates how many standard deviations a particular compound is above or below the mean of the plate, and is calculated as follows: Z-Score = (x−μ)/δ, where x is the raw signal, μ is the mean signal of all the compound-containing wells of one plate, and δ is the standard deviation of all compound-containing wells of one plate. Primary hits were identified by calculating a Z-score for each compound and applying hit selection criteria: A compound was considered a hit if one replicate had a Z-Score>3 and the other replicate had a Z-Score of >2.5.

In cell viability assays, the relative metabolic activity of ATP was calculated according to the following formula: % ATP = (x/μ_neg_)*100, where x is the raw signal for each compound, and μ_neg_ is the mean signal for the negative control (DMSO-treated cells). This calculation normalizes DMSO-treated cells (negative control) to 100% ATP activity.

Analysis of the confirmation screen assay was done using a fold induction calculation to normalize each compound signal. The following formula was used: Fold Induction = x/μ_neg_ where x is the raw signal for each compound, and μ_neg_ is the mean signal for the negative control (either DMSO-treatment for compound treated cells, or DMSO-treated infection (rPR8, WSN, or VSV) for compound-treated infections). Confirmed hits were selected by calculating the fold induction for each compound and applying selection criteria: A compound was confirmed if it displayed a dose-dependent effect with a minimum 5 fold induction, either in the presence or absence of influenza virus (rPR8) infection.

### Cell viability assay

The CellTiterGlo Cell Viability Assay (Promega) was used to detect ATP levels as a function of cell viability, according to manufacturer's specifications. Cells were seeded into 96-well plates (7500 cells/well) and incubated at 37°C, 5% CO_2_ for 24 hours. Culture medium was then replaced with 50 µL of fresh medium containing compound (serially diluted), and this was further incubated for 48 hours. Luciferase production was measured by adding 50 µL of CellTiterGlo reagent (diluted 2 fold with PBS) to each well, and the luminescence signal was read using the Beckman plate reader. Relative metabolic activity of ATP was calculated as described in the ‘data analysis’ section.

### Confirmation screen assay

Confirmation of the primary hits was done with re-ordered compounds from commercial vendors, as described in the ‘small molecular weight compounds’ section. MDCK IFNβ-luciferase cells were plated into 96-well plates with 50 µL of cells (3×10^5^ cells/mL), and incubated for 20 hours. Ten serial dilutions of compounds were made in 100% DMSO, starting from the highest non-toxic soluble concentration. Serial dilutions in DMSO were further diluted 200 fold in post-infection medium (DMEM supplemented with 0.1% FBS, 1% P/S, 0.5% BSA, 1 µg/mL TPCK) to obtain a final 0.5% DMSO concentration. These were then added to cells in a 96-well plate excluding the outside wells (60 wells total), and incubated for 2 hours prior to infection. The top half of the plate (30 wells) was left uninfected, whereas the bottom half was infected with rPR8 (MOI = 10), WSN (MOI = 1), or VSV (MOI = 0.01) by adding 10 µL of the virus inoculum (PBS supplemented with 0.5% BSA and 1% P/S) directly to the medium. Each condition was tested in triplicate. Mock plates infected with rPR8 NS1-113 or without infection were used as controls. Infection proceeded at 37°C, 5% CO_2_ for 18 hours prior to measuring luciferase production by adding 50 µL of Bright-Glo reagent (diluted 3 fold with PBS) to each well. The luminescence signal was measured using the Beckman plate reader. Fold induction was calculated as described in the ‘data analysis’ section.

### Infection of cells in the presence of inhibitors

To test the effects of compounds on multi-cycle influenza virus replication, cells were seeded into 6-well plates at 5×10^5^ cells/well or 12-well plates at 2×10^5^ cells/well and incubated at 37°C, 5% CO_2_ for 24 hours. Cells were then washed with PBS and infected with the indicated virus in PBS/0.5% BSA infection medium at MOI = 0.01 (unless otherwise indicated) in a 200 µL inoculum. After 1 hour incubation at room temperature, the virus inoculum was aspirated, cells were washed with PBS, and post-infection medium (with 1 µg/mL TPCK) containing compound was added to cells and incubated for 48 hours during influenza virus and SinV infections, and for 18 hours during VSV.. Viral titers of influenza viruses were determined by standard plaque assay in MDCK cells. Viral titers of VSV and SinV were determined by standard plaque assay in VERO cells.

To test the effect of ASN2 on IFN induction, cells were infected at an MOI = 1, and incubated with post-infection medium containing compound for 24 hours. Final concentration of ASN2 was 50 µM, unless indicated.

### RNA isolation

RNA extraction of cells for subsequent qRT-PCR analysis was performed using the RNeasy Mini Kit (Qiagen, Valencia, CA) according to manufacturer's specifications. RNA extraction for deep sequencing was performed using the TRIzol Reagent (Invitrogen) according to manufacturer's specifications. RNA extraction of viral RNA was performed using the QIAamp Viral RNA Mini Kit (Qiagen) according to manufacturer's specifications. RNA yields were evaluated using a Nanodrop spectrophotometer (Nanodrop technologies, Wilmington, DE).

### RT-PCR and quantitative RT-PCR (qRT-PCR)

A two-step qRT-PCR was performed to analyze cellular and viral genes. First-strand synthesis of isolated RNA was done by reverse transcription using Superscript III reverse transcriptase (Invitrogen), Random Primers (Invitrogen), and RNase Out (Invitrogen), according to manufacturer's specifications. Resulting cDNA was diluted to 2–5 ng/µL in PCR-grade water and used for quantitative PCR using LightCycler 480 SYBR Green I Master reagent (Roche), and the LightCycler 480 instrument (Roche), according to manufacturer's specifications. Analysis of results were done using the 2^−ΔΔCT^ (two delta delta CT) method [44]. We calculated ΔΔC_T_ values over replicates using α-tubulin as the endogenous housekeeping gene and DMSO-treated uninfected (MOCK) or DMSO-treated infected samples as the calibrator in respective experiments. Values represent the fold difference for each condition as compared with DMSO-treated uninfected or DMSO-treated infected samples. Error bars indicate ± standard deviation of fold induction.

For sequencing of different influenza A virus segments of the ASN2 resistant virus and recombinant influenza viruses, a one-step RT-PCR was performed using the Superscript III One-Step RT-PCR System with Platinum Taq High Fidelity (Invitrogen), according to manufacturer's specifications.

### Antiviral bioassay

To test the biological activity of cytokines released during ASN2 treatment of infected cells, an antiviral bioassay was performed as previously described with some differences [45]. A549 cells were plated into 96-well plates with 50 µL of cells (1×10^4^ cells/well), and incubated at 37°C, 5% CO_2_ for 24 hours. The culture medium was replaced with post-infection medium containing compound and 1 µg/mL TPCK. After 2 hours incubation, these were infected with rPR8 (MOI = 10) or WSN (MOI = 1) by adding 10 µL of virus inoculum directly into the medium. Sendai virus (SeV) infection was used as a positive control for interferon induction. At 18 hours post infection, supernatants were harvested and virus present in the supernatant was UV inactivated by placing the 96-well plate in a UV chamber delivering 200 J/cm^2^. Two-fold dilutions of the inactivated supernatants were added to fresh VERO cells previously seeded in 96-well plates. Following a 24 hour incubation, VERO cell supernatants were aspirated, and cells were infected with 50 µL NDV-GFP diluted in OptiMEM. (The concentration of NDV-GFP used was previously determined to yield 90% GFP expression at 24 hours). GFP was visualized 24 hours post infection by fluorescence microscopy and quantified using the Beckman plate reader.

Percent inhibition of NDV-GFP induced by the supernatants was calculated using the following formula: % inhibiton = 100−(x/μ_neg_ *100), where x is the raw signal for each treatment, μ_neg_ is the mean signal for the negative control (MOCK: untreated and NDV-GFP infected cells).

### Deep sequencing

Total RNA was isolated from infected cells treated with ASN2 or DMSO as previously described in the ‘RNA isolation’ section. Processing of the RNA and subsequent sequencing was performed by the Mount Sinai sequencing facility using the Illumina Genome Analyzer (Illumina, San Diego, CA). Briefly, mRNA was isolated using oligo(dT)-coated beads, and subsequently fragmented using the Covaris RNA Shearing kit (Covaris, Woburn, Massachusetts). After precipitation of RNA, first strand cDNA synthesis was performed with Superscript II reverse transcriptase (Invitrogen), Random Primers (Invitrogen), and RNase Inhibitor (Invitrogen). Second strand cDNA synthesis, end repair, A-tailing, and adaptor ligations were done using the Illumina Sample Prep Kit (Illumina). Elution of cDNA fragments and gel purification was done using the MinElute Gel Extraction Kit (Qiagen). A 200–300 bp gel piece was cut prior to isolation of cDNA using the QIA Quick Gel Extraction Kit (Qiagen). PCR amplification and purification of fragments were done prior to sequencing on the Illumina platform.

Sequences were mapped to both human and influenza virus genomes. Relative abundance was normalized to the values obtained for the GAPDH mRNA, and average ratios between infected cells treated with ASN2 or DMSO were calculated. The ratios represent fold induction of ASN2 treatment over DMSO treatment.

### Western blot

Cells were washed twice with cold PBS and lysed in a 0.5% Nonidet P-40 lysis buffer. Samples were run on 4–20% precast gradient gels (Bio-Rad, Hercules, CA) and transferred onto polyvinylidenefluoride (PVDF) membranes (GE Healthcare, Buckinghamshire, UK). GAPDH, PB1, NP, NS1, M1, and M2 proteins were detected using the rabbit polyclonal anti-glyceraldehyde 3-phosphate dehydrogenase (GAPDH) antibody (Sigma), a rabbit polyclonal PB1 antibody (generated by peptide immunization), a mouse monoclonal NS1 antibody (clone 1A7, Mount Sinai Hyridoma Facility), a mouse monoclonal NP antibody (clone 28D8, Mount Sinai Hyridoma Facility), a mouse monoclonal M1 and M2 antibody (clone E10, Mount Sinai Hyridoma Facility), and horseradish peroxidase-conjugated secondary antibodies (GE Healthcare).

### Animal experiments

#### Ethics statement

This study was carried out in strict accordance with the recommendations in the Guide for the Care and Use of Laboratory Animals of the National Institutes of Health. The protocol was approved by the Mount Sinai School of Medicine Institutional Animal Care and Use Committee (IACUC) (Animal Assurance No. A3111-01). Mice were euthanized at the end of the experiment according to these guidelines and all efforts were made to minimize suffering.

Six to eight week old female BALB/c mice (Jackson Laboratory, Bar Harbor, ME) were separated into groups of 9 mice. Pretreatment was done by administering one dose of ASN2 (33.3 mg/kg), oseltamivir (0.5 mg/kg) or solvent (33% DMSO, 33% PBS, and 33% Labrafil (Gattefosse, Paramus, NJ)) intraperitoneally (IP) 8 hours and 1 hour prior to infection. Mice were anesthetized with a mixture of ketamine and xylazine IP, and infected intranasally with influenza A/WSN/33 virus (5MLD_50_). ASN2 (100 mg/kg/day), oseltamivir (1.5 mg/kg/day), and respective solvent treatments proceeded every 8 hours for 8 days. Bodyweight and survival was monitored daily. Three mice from each group were euthanized on days 3 and 8 post infection and viral lung titers were determined by plaques assay.

### In vitro liver microsome assay

This analysis was performed by Cyprotex. Pooled Balb/C mouse liver microsomes (final protein concentration 0.5 mg/ml), 0.1 M phosphate buffer pH 7.4 and ASN2 (final concentration 3 µM in 0.25% DMSO) were pre-incubated at 37°C prior to addition of NADPH (final concentration 1 mM) to initiate the reaction. Controls included samples that lacked NADPH and samples that contained control compounds to be used as internal standards. Samples were incubated for 0, 5, 15, 30 and 45 min. The reactions were stopped by the addition of 50 µl methanol. Protein was precipitated by centrifugation at 25000 rpm for 20 min at 4°C and the supernatants were analyzed by LC-MS/MS to detect and quantify the compounds at each time-point. Intrinsic clearance and half-life were calculated from a plot of the peak area ratio (ASN2 peak area/internal standard peak area) against time.

### IFNβ reporter assays

For IFNβ reporter assays, A549 cells were transfected with lipofectamine LTX (Invitrogen). Transfections were done in 24-well plates at cell concentrations of 2×10^5^ cells/well for A549 cells, at a lipid∶DNA ratio of 2∶1. 125 ng of IFNβ-LUC reporter, 50 ng pRL-TK reporter, and 325 ng of empty pCAGGS vector or pCAGGS-NS1 were transfected into one well using 1 µL transfection reagent in 75 mL Opti-MEM. Incubation of lipid and DNA was done at room temperature for 30 minutes prior to adding the transfection complex directly to cells containing regular growth medium. 24 hours post transfection, cells were washed and infected with influenza viruses (as indicated) at an MOI = 1 for 1 hour prior to addition of post infection medium containing DMSO or ASN2. Infections proceeded for 24 hours prior to assaying for luciferase activity using the Dual-Glo Luciferase Assay kit (Promega), according to manufacturer's specifications.

### Plaque-reduction assay

Cells were seeded into 6-well plates until 100% confluency was achieved. The indicated viruses were diluted 10^4^ to 10^6^ fold from their stock concentration. Each dilution was used to infect one 6-well plate at a 200 µL inoculum, and incubated at room temperature for 1 hour. Virus inoculum was aspirated and plaque overlay medium containing DMSO or ASN2 (50 µM and 25 µM) was added to cells, which were incubated at 37°C, 5% CO_2_ for 48–72 hours. Plaques were visualized by immunostaining with an NP antibody and horseradish peroxidase-conjugated secondary antibody.

### Influenza virus minigenome assays

For influenza A virus minigenome reporter assays, A549 cells were transfected with lipofectamine LTX (Invitrogen). Transfections were done in 24-well plates at cell concentrations of 2×10^5^ cells/well for A549 cells, at a lipid∶DNA ratio of 2∶1. 75 ng of WSN NP_LUC reporter, 50 ng pRL-TK reporter, 50 ng of WSN PB1, WSN PB2, and WSN PA expression plasmids, and 100 ng WSN NP expression plasmid (or empty vector for negative control) were co-transfected with 0.75 µL transfection reagent in 0.75 mL Opti-MEM. Incubation of lipid and DNA was done at room temperature for 30 minutes prior to adding the transfection complex directly to cells containing post infection medium (without TPCK) supplemented with ASN2 or DMSO. 24 hours post transfection, cells were lysed and luciferase production was measured with the Dual-Glo Luciferase Assay kit (Promega), according to the manufacturer's specifications. For the influenza B virus minigenome assay a plasmid encoding the chloramphenicol acetyl transferase (CAT) gene flanked by the non-coding regions from the NS segment of influenza B/Lee/40 virus (pPOLI-B/Lee/40 NS-CAT) was used [46]. The PB1, PB2, PA and NP expression constructs were derived from influenza B/Yamagata/88 virus. CAT expression was assayed by ELISA (Roche).

### Primer extension assay

A549 cells were seeded into 10 cm dishes (Corning) at 2.5×10^6^ cells/dish in regular growth medium and incubated at 37°C, 5% CO_2_ for 24 hours. Cell culture medium was then replaced with post-infection medium (with 1 µg/mL TPCK) containing DMSO, ASN2 (50 µM, 25 µM, and 12.5 µM) or ribavirin (100 µM) for 4 hours prior to infection. Cells were then washed with PBS and infected with WSN in PBS/0.5% BSA infection medium (MOI = 5) in a 1 mL inoculum. After 1 hour incubation at room temperature, virus inoculum was aspirated, cells were washed with PBS, and post-infection medium (with 1 µg/mL TPCK) containing the respective compounds was added to cells and incubated for 6 hours. RNA was extracted using the RNeasy Mini Kit (Qiagen). Primer labeling, primer extension reaction, and gel analysis of products were performed using the Primer Extension System – AMV Reverse Transcriptase kit (Promega) according to the manufacturer's specifications. Primers used for primer extension can be found in [34]. Resulting blots were visualized with the phosphorimager Typhoon Variable Mode Imager (GE Heathcare, Piscataway, NJ) and quantified using ImageJ software.

## Supporting Information

Figure S1 Schematic of high-throughput compound screen. In the primary screen (1), MDCK IFNβ-luciferase cells were seeded into 384-well plates and incubated for 20 hours prior to pin transfer of compounds. Two hours later, wells were infected with influenza A/PR/8/34 virus (MOI = 10) for 18 hours before luciferase activity was measured. Two columns in each plate were reserved for the controls and did not contain compounds. The second to last column was infected with wild type PR8 virus only (negative control) and the last column was infected with PR8 NS1-113 virus only (positive control). Primary hits were identified upon calculation of a Z-Score for each compound and application of the hit criteria as indicated. The secondary screen (2) was performed with 250 compounds out of the 264 identified hits. This screen was done in a 96-well format, and included the secondary assays shown. Confirmed hits (27) were selected based on the confirmation criteria indicated. These hits were divided into two groups: compounds that induce IFNβ independently of virus infection, and compounds that required virus infection to induce IFNβ. ASN2 was selected as the lead compound (3) for further evaluation.
(TIF)

Figure S2 Replication of influenza A virus is required for induction of interferon upon ASN2 treatment. (A) qRT-PCR analysis of IFNβ and ISG56 mRNA in A549 cells infected with A/WSN/33 (MOI = 1) and treated with decreasing concentrations of ASN2 (50-12.5 µM) for 24 hours. (B) qRT-PCR analysis of IFNβ and ISG56 mRNA in A549 cells infected with UV-inactivated A/WSN/33 (MOI = 1) and treated with decreasing concentrations of ASN2 (50-12.5 µM) for 24 hours. IFNβ treatment (50 IU/mL) was used as a positive control. Values were normalized to α-tubulin for each sample and are represented as fold induction over uninfected DMSO-treated sample. Error bars reflect standard deviation of fold change.
(TIF)

Figure S3 ASN2 preferentially inhibits the production of viral mRNA from smaller influenza virus genome segments. (A) Deep sequencing analysis of influenza virus mRNA in A549 cells infected with A/WSN/33 (MOI = 1) and treated with DMSO or ASN2 (50 µM) for 24 hours. Y axis represents the total number of reads for each particular sequence (50 nt long), and X axis represents the position of each read in the viral genome. (B) Analysis of sequence reads from part A. Values were normalized to total reads for each sample and are represented as ASN2/DMSO ratio.
(TIF)

Figure S4 ASN2 is a unique interferon-inducing antiviral compound. qRT-PCR analysis of IFNβ mRNA in A549 cells infected with influenza A/WSN/33 virus (MOI = 1) and treated with ASN2 (50 µM) or A3 (10 µM) for 24 hours. Values were normalized to α-tubulin for each sample and are represented as fold induction over uninfected DMSO-treated sample. Error bars reflect standard deviation of fold change.
(TIF)

Table S1 Transcriptional profile of genes induced by ASN2 in influenza virus infected cells.
(TIF)

Table S2 Amino acid conservation at position 499 of the influenza A virus PB1 protein.
(TIF)
