# Supplementary figures and images for: A Novel Small Molecule Inhibitor of Influenza A Viruses that Targets Polymerase Function and Indirectly Induces Interferon
Source: PLoS Pathog. 2012 Apr 26;8(4):e1002668. doi: 10.1371/journal.ppat.1002668 (PMC3343121; doi:10.1371/journal.ppat.1002668)

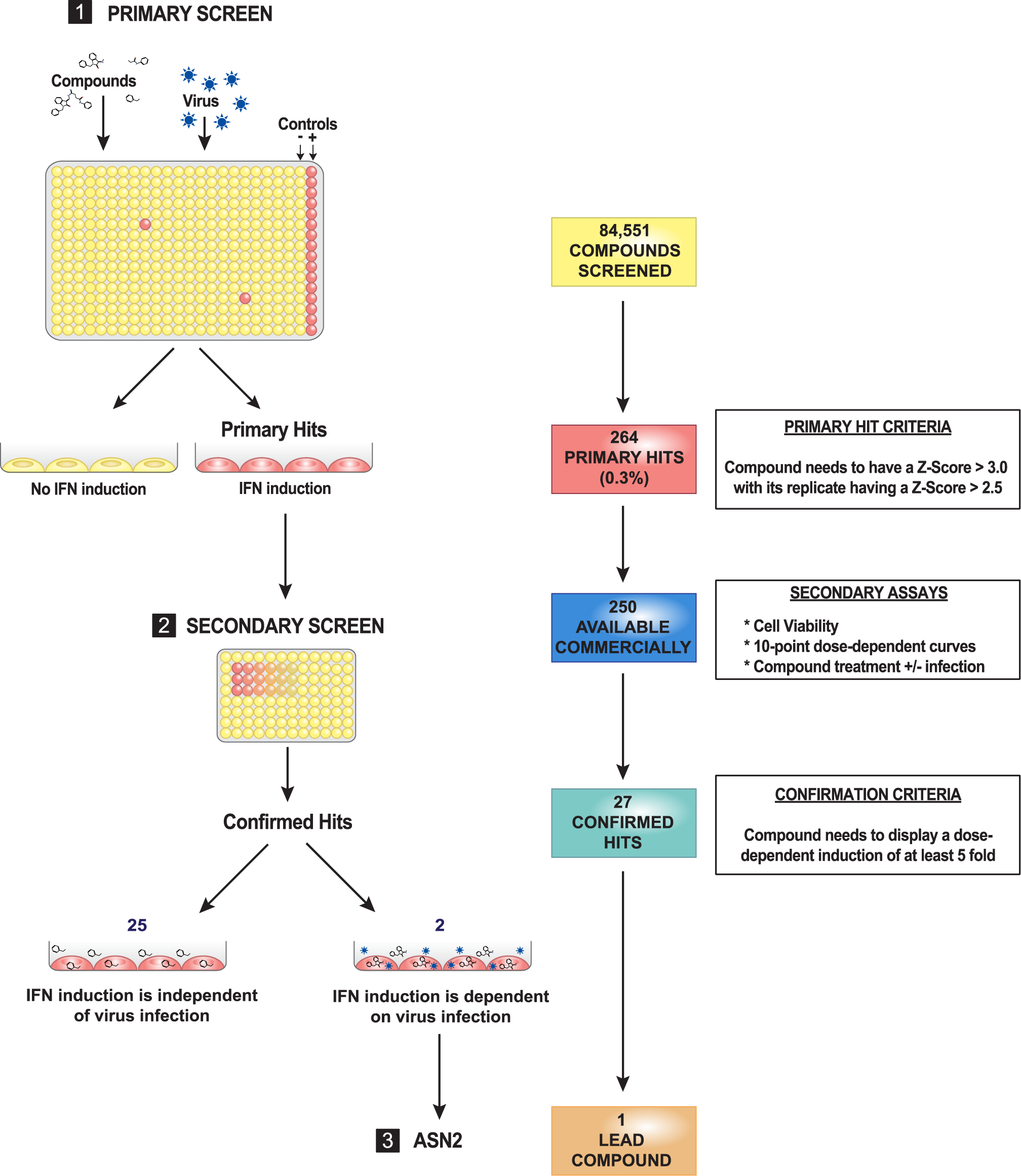

Supplement: Figure S1 — Schematic of high-throughput compound screen. In the primary screen (1), MDCK IFNβ-luciferase cells were seeded into 384-well plates and incubated for 20 hours prior to pin transfer of compounds. Two hours later, wells were infected with influenza A/PR/8/34 virus (MOI = 10) for 18 hours before luciferase activity was measured. Two columns in each plate were reserved for the controls and did not contain compounds. The second to last column was infected with wild type PR8 virus only (negative control) and the last column was infected with PR8 NS1-113 virus only (positive control). Primary hits were identified upon calculation of a Z-Score for each compound and application of the hit criteria as indicated. The secondary screen (2) was performed with 250 compounds out of the 264 identified hits. This screen was done in a 96-well format, and included the secondary assays shown. Confirmed hits (27) were selected based on the confirmation criteria indicated. These hits were divided into two groups: compounds that induce IFNβ independently of virus infection, and compounds that required virus infection to induce IFNβ. ASN2 was selected as the lead compound (3) for further evaluation. (TIF) [file ppat.1002668.s001.tif]

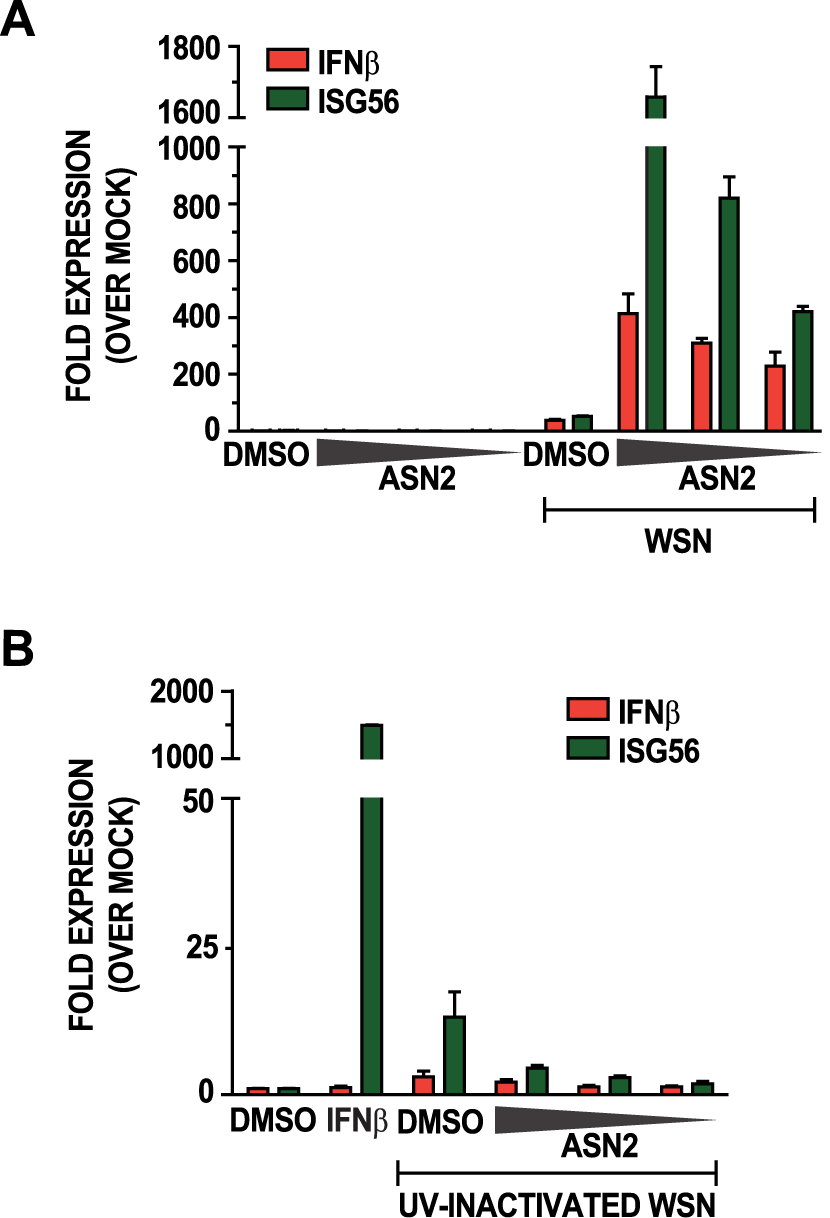

Supplement: Figure S2 — Replication of influenza A virus is required for induction of interferon upon ASN2 treatment. (A) qRT-PCR analysis of IFNβ and ISG56 mRNA in A549 cells infected with A/WSN/33 (MOI = 1) and treated with decreasing concentrations of ASN2 (50-12.5 µM) for 24 hours. (B) qRT-PCR analysis of IFNβ and ISG56 mRNA in A549 cells infected with UV-inactivated A/WSN/33 (MOI = 1) and treated with decreasing concentrations of ASN2 (50-12.5 µM) for 24 hours. IFNβ treatment (50 IU/mL) was used as a positive control. Values were normalized to α-tubulin for each sample and are represented as fold induction over uninfected DMSO-treated sample. Error bars reflect standard deviation of fold change. (TIF) [file ppat.1002668.s002.tif]

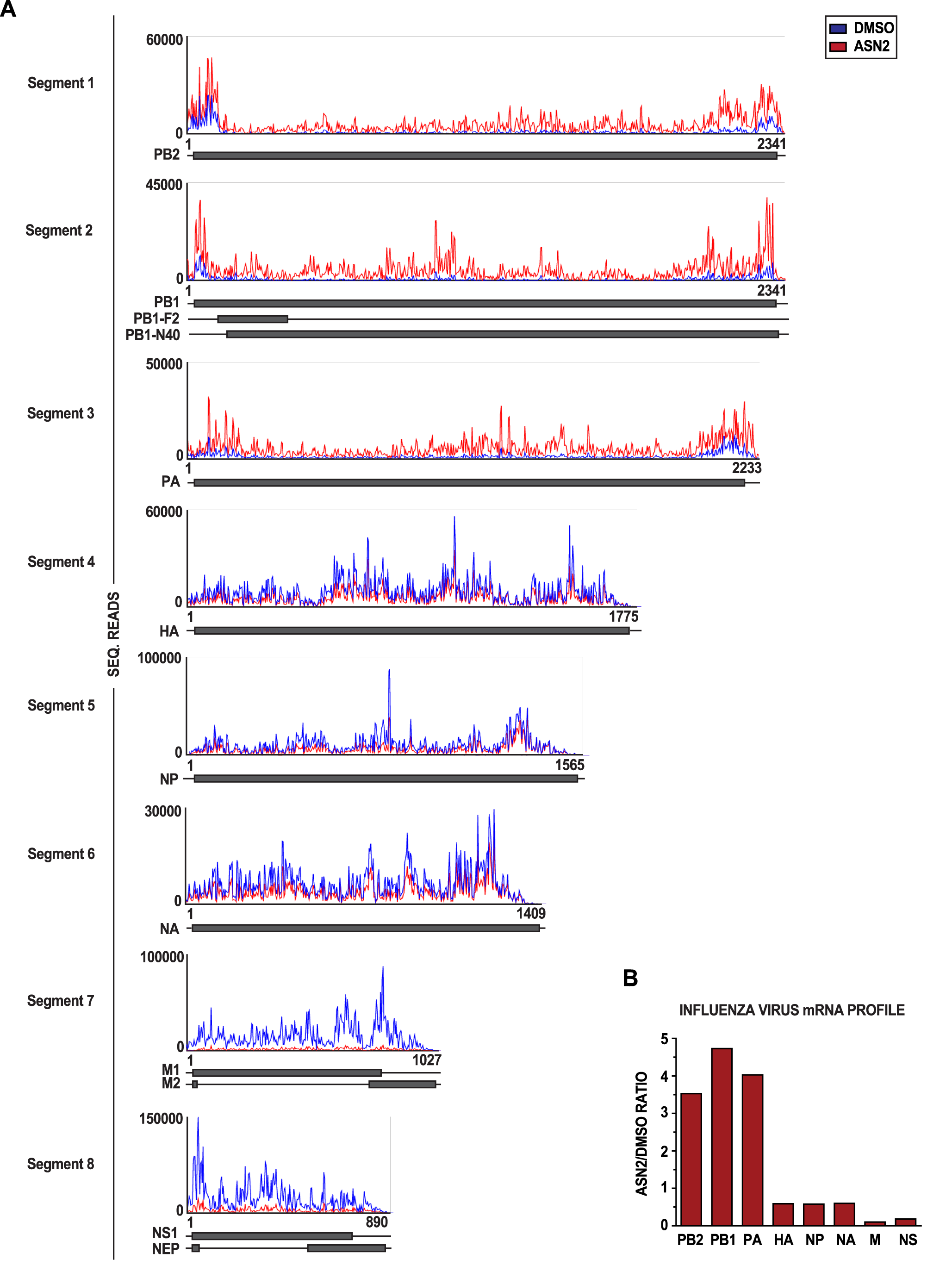

Supplement: Figure S3 — ASN2 preferentially inhibits the production of viral mRNA from smaller influenza virus genome segments. (A) Deep sequencing analysis of influenza virus mRNA in A549 cells infected with A/WSN/33 (MOI = 1) and treated with DMSO or ASN2 (50 µM) for 24 hours. Y axis represents the total number of reads for each particular sequence (50 nt long), and X axis represents the position of each read in the viral genome. (B) Analysis of sequence reads from part A. Values were normalized to total reads for each sample and are represented as ASN2/DMSO ratio. (TIF) [file ppat.1002668.s003.tif]

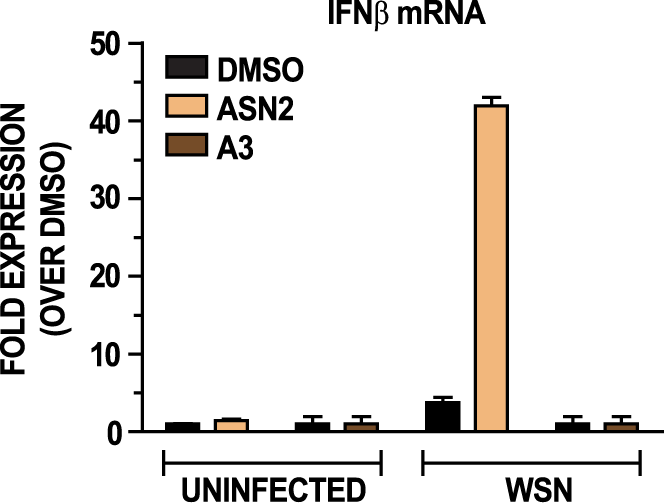

Supplement: Figure S4 — ASN2 is a unique interferon-inducing antiviral compound. qRT-PCR analysis of IFNβ mRNA in A549 cells infected with influenza A/WSN/33 virus (MOI = 1) and treated with ASN2 (50 µM) or A3 (10 µM) for 24 hours. Values were normalized to α-tubulin for each sample and are represented as fold induction over uninfected DMSO-treated sample. Error bars reflect standard deviation of fold change. (TIF) [file ppat.1002668.s004.tif]

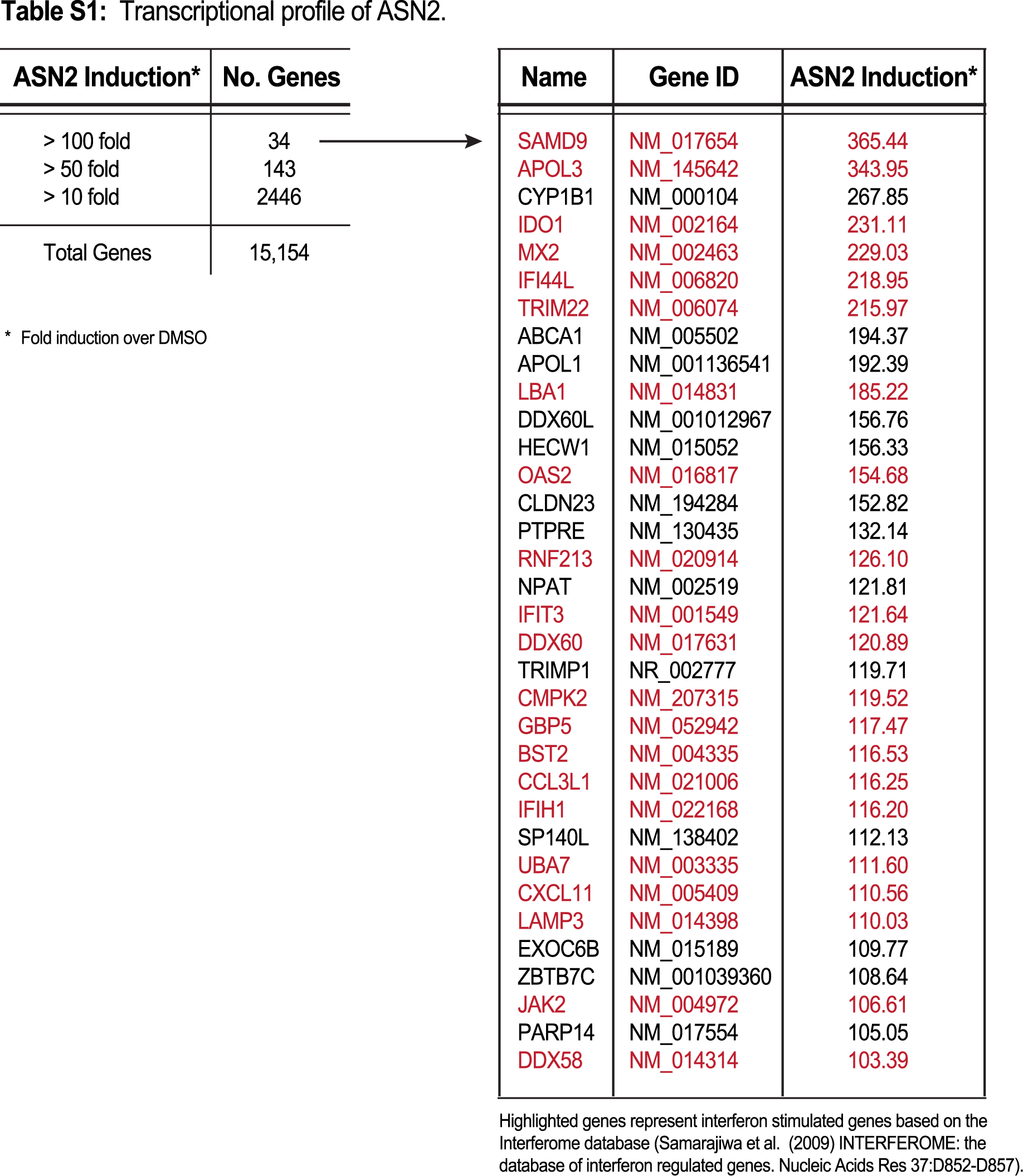

Supplement: Table S1 — Transcriptional profile of genes induced by ASN2 in influenza virus infected cells. (TIF) [file ppat.1002668.s005.tif]

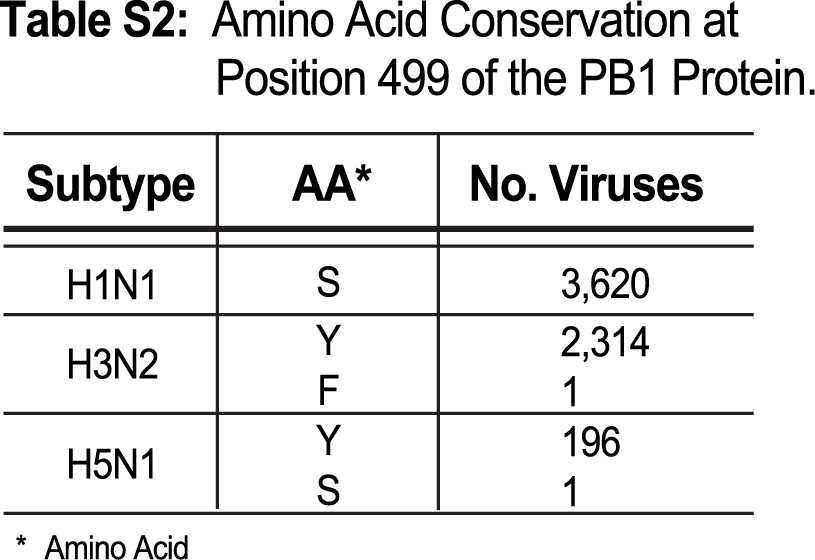

Supplement: Table S2 — Amino acid conservation at position 499 of the influenza A virus PB1 protein. (TIF) [file ppat.1002668.s006.tif]
